# Diversity, dynamics, direction, and magnitude of high-altitude migrating insects in the Sahel

**DOI:** 10.1038/s41598-020-77196-7

**Published:** 2020-11-25

**Authors:** Jenna Florio, Laura M. Verú, Adama Dao, Alpha S. Yaro, Moussa Diallo, Zana L. Sanogo, Djibril Samaké, Diana L. Huestis, Ousman Yossi, Elijah Talamas, M. Lourdes Chamorro, J. Howard Frank, Maurizio Biondi, Carsten Morkel, Charles Bartlett, Yvonne-Marie Linton, Ehud Strobach, Jason W. Chapman, Don R. Reynolds, Roy Faiman, Benjamin J. Krajacich, Corey S. Smith, Tovi Lehmann

**Affiliations:** 1 Laboratory of Malaria and Vector Research, NIAID, NIH, Rockville, MD USA; 2 Malaria Research and Training Center (MRTC), Faculty of Medicine, Pharmacy and Odonto-Stomatology, Bamako, Mali; 3 Systematic Entomology Laboratory - ARS, USDA C/O Smithsonian Institution, National Museum of Natural History, Washington, DC USA; 4 Florida Department of Agriculture and Consumer Services, Division of Plant Industry, Gainesville, FL USA; 5 Entomology and Nematology Department, University of Florida, Gainesville, FL USA; 6 Department of Life, Health, and Environmental Sciences, University of L’Aquila, L’Aquila, Italy; 7 Institute of Applied Entomology, Beverungen, Germany; 8 Department of Entomology and Wildlife Ecology, University of Delaware, Newark, DE USA; 9 Earth System Science Interdisciplinary Center, University of Maryland, College Park, MD USA; 10 Walter Reed Biosystematics Unit, Smithsonian Institution Museum Support Center, Suitland, MD USA; 11 Department of Entomology, Smithsonian Institution, National Museum of Natural History, Washington, DC USA; 12 Centre for Ecology and Conservation, and Environment and Sustainability Inst., University of Exeter, Penryn, Cornwall UK; 13 College of Plant Protection, Nanjing Agricultural University, Nanjing, People’s Republic of China; 14 Natural Resources Institute, University of Greenwich, Chatham, Kent, ME4 4TB UK; 15 Rothamsted Research, Harpenden, Hertfordshire, AL5 2JQ UK; 16 American Museum of Natural History, New York, NY USA

**Keywords:** Ecology, Zoology

## Abstract

Long-distance migration of insects impacts food security, public health, and conservation–issues that are especially significant in Africa. Windborne migration is a key strategy enabling exploitation of ephemeral havens such as the Sahel, however, its knowledge remains sparse. In this first cross-season investigation (3 years) of the aerial fauna over Africa, we sampled insects flying 40–290 m above ground in Mali, using nets mounted on tethered helium-filled balloons. Nearly half a million insects were caught, representing at least 100 families from thirteen orders. Control nets confirmed that the insects were captured at altitude. Thirteen ecologically and phylogenetically diverse species were studied in detail. Migration of all species peaked during the wet season every year across localities, suggesting regular migrations. Species differed in flight altitude, seasonality, and associated weather conditions. All taxa exhibited frequent flights on southerly winds, accounting for the recolonization of the Sahel from southern source populations. “Return” southward movement occurred in most taxa. Estimates of the seasonal number of migrants per species crossing Mali at latitude 14°N were in the trillions, and the nightly distances traversed reached hundreds of kilometers. The magnitude and diversity of windborne insect migration highlight its importance and impacts on Sahelian and neighboring ecosystems.

## Introduction

Migration is key to individual reproductive success, population abundance and range, community composition, and thus, habitat function across the biosphere^1,2^. We follow the definition of migration as persistent movements unaffected by immediate cues for food, reproduction, or shelter, with a high probability of relocating the animal in a new environment^1–3^. Long-distance insect migration influences food security^2,4–9^, public health^10–15^, and ecosystem vigor^16,17^. Over the past decades, knowledge of the migration of a handful of large insects (> 40 mg) provided insights into migratory routes and the underlying physiology and ecology of migration with implications ranging from pest control to conservation^18–21^. Radar studies have revealed the magnitude of insect migration, highlighting its role in ecosystem biogeochemistry via the transfer of micronutrients by trillions of insects moving annually in Europe^22,23^. Yet, radar studies seldom provide species-level information^24^, which is needed to discern the adaptive strategies, drivers, processes, and impacts of long-distance migration of the vast majority of the species^22,25^. Ideally, addressing these issues requires tracking insects over hundreds of kilometers, a task that remains beyond reach for most species due to their small size, speed, and flight hundreds of meters above ground level (agl)^26^. Given migration’s pervasive and critical role, knowledge of the species identity, sources, routes, destinations, schedules, and impacts would be especially valuable for sub-Saharan Africa, with its growing human population, nutritional demands, public health problems, and conservation challenges. Past migration studies in Africa focused on a handful of crop pests such as grasshoppers^4,7,27,28^ and the African armyworm^24,29^, yet as recently demonstrated by the diversity of mosquitoes among high-altitude migrants^15^, the scope and impacts of African insect migration represent major gaps in our knowledge. We contend that monitoring of insect migrants in Africa will not only fill that gap, but will lead to effective and comprehensive solutions inspired by the locust and armyworm monitoring and control programs^4,28,29^, which fit well with the One Health paradigm^30^. Accordingly, a longitudinal, systematic, comparative study on the high-altitude migration of multiple species of insects in the same region would be useful to gauge the species composition, regularity, dynamics, and directionality, which are fundamental to understand these movements’ predictability, sources, and impacts. Specifically, we focused on the following questions: Which taxa are the dominant windborne migrants? Do species migrate regularly every year? Within a year, does migration occur rarely, under specific weather conditions, or throughout the season? What are the prominent flight directions, how variable are they within and between species, and does the direction change with the season? Using nets mounted between 40 and 290 m agl on tethered helium balloons, we undertook a three-year survey of flying insects in central Mali. Here, focusing on a dozen collected insect species—representing broad phylogenetic groups and ecological “guilds”—we identify variation in migration patterns, and infer underlying strategies.

## Results

In total, 461,100 insects were collected on 1,894 panels between 2013 and 2015. Sorting of 4,824 specimens from 77 panels (between 40 and 290 m agl) revealed a diverse assembly representing thirteen orders (Fig. 2a and Table 1). Members of the Coleoptera dominated these collections at 53%, followed by Hemiptera (27%)—especially Auchenorrhyncha (18.5%), Diptera (11%), and Hymenoptera and Lepidoptera at 4% each, together accounting for > 99% of the insects collected (Table 1). Additional specimens were identified totaling 100 insect families (Tables 1 and S1).

**Table 1:** Overall diversity of insects collected in aerial samples (40–290 m agl) as reflected by insect order composition (see also Fig. 2). The insect families sent for identification by taxonomists represent a small fraction of the predicted total diversity (see text). Orders represented by 1–2 specimens (Blattodea, Thysanoptera, Megaloptera, Psocoptera, and Phasmatodea) are not shown.

Order	Percent	Families identified	Families
Coleoptera	53.3	Aderidae, Anthicidae, Attelabidae, Bostrichidae, Brentidae, Carabidae, Chrysomelidae, Coccinellidae, Curculionidae, Dytiscidae, Elateridae, Erorhinidae Hydrophilidae, Mordellidae, Nitidulidae, Phalacridae, Scarabaeidae, Staphylinidae	18
Hemiptera (Heteroptera)	8.2	Berytidae, Corixidae, Cydnidae, Geocoridae, Gerridae, Hydrometridae, Lygaeidae, Miridae, Nabidae, Notonectidae, Oxycarenidae, Pentatomidae, Pyrrhocoridae, Reduviidae, Rhopalidae, Rhyparochromidae, Stenocephalidae, Tingidae, Veliidae	19
Hemiptera (Homoptera)	19	Aphididae, Cicadellidae, Delphacidae, Flatidae, Ricaniidae	5
Diptera	11.2	Anthomyiidae, Calliphoridae, Cecidomyiidae^b^, Ceratopogonidae^b^, Chironomidae^b^, Chloropidae, Culicidae^b^, Curtonotidae, Diopsidae, Dolichopodidae, Drosophilidae, Ephydridae, Lauxaniidae, Limoniidae^b^, Lonchaeidae, Milichiidae, Muscidae, Mycetophilidae^b^, Phoridae, Pipinculidae, Platystomatidae, Rhiniidae, Sepsidae, Simuliidae, Tachinidae, Tephritidae, Tipulidae, Ulidiidae	28
Hymenoptera	4	Apidae, Bethylidae, Braconidae, Chalcididae, Chrysididae, Crabronidae, Diapriidae, Dryinidae, Eulophidae, Eupelmidae, Eurytomidae, Figitidae, Formicidae, Ichneumonidae, Megachilidae, Pompilidae, Rhopalosomatidae, Scelionidae, Sphecidae	19
Lepidoptera	3.9	Gelechiidae^b^, Nolidae^b^	2
Orthoptera	0.3	Acrididae, Gryllidae, Pyrgomorphidae, Tetrigidae, Tettigonidae, Trigonidiidae	6
Neuroptera	0.2	Chrysopidae, Mantispidae, Myrmeleontidae	3
Total			100

^a^ In total, 4,824 insects from 77 sticky nets (panels) were used to estimate the order composition.

The full collection awaits additional study and the authors would be pleased to hear from readers who might be interested in undertaking further study of particular taxa.

^b^ Identified through DNA barcoding correlations by Dr. Yvonne-Marie Linton.

**Figure 1:**
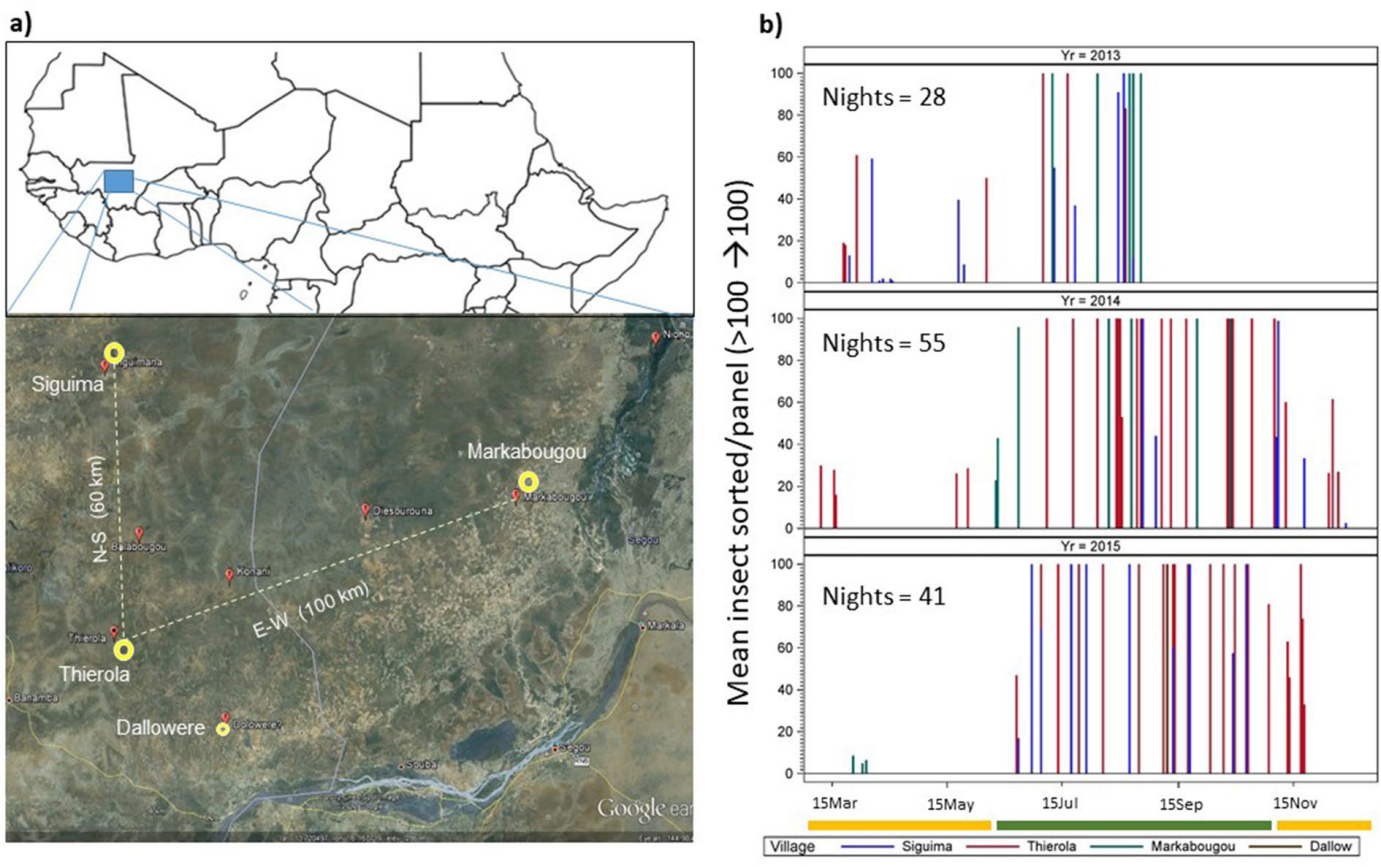
(**a**) Map of study area (Map data: Google, Maxar Technologies) under a schematic map of Africa above the equator. The base map was generated using the ggplot2 package in R^74^, under a GPL-2 license. Aerial collection sites are shown in yellow with distance between them (the small symbol of Dallowere indicates that only two sampling nights in Dallowere were included in the present study). (**b**) Sampling effort of high-altitude flying insects by year. Needles represent sampling nights (by village: color) extending up to 100 insects per panel (actual number of insects can exceed 2000). Dry and wet seasons are indicated by yellow and green bands, respectively, under the x axis. Note: no sampling was done during January–February.

Thirteen species representing diverse phylogenetic and ecological groups were identified and counted from subsamples consisting of a total of 25,188 specimens. These were subsamples (see Methods) of 58,706 insects captured on 222 panels over 125 aerial collections, carried out over 96 sampling nights, in one or more of the Sahelian villages (Fig. 1).

Control panels were examined to determine if insects were inadvertently trapped near the ground as the nets were raised and lowered: a total of 564 insects were captured on 508 control panels compared with 58,706 captured on the 222 experimental (standard) panels. The control panels spent 3–5 min above 30 m and thus could be expected to contain ~ 0.5% of a normal panel that remained at high altitude for 14 h, assuming aerial density of the insects remained constant over that time. To assess if insects were intercepted below 30 m agl, we tested if the mean panel density (Methods) of each taxon on the control panel was (i) significantly lower than corresponding mean on standard panel, and (ii) that it was not significantly higher than the equivalent of a 4-min aerial nightly sampling with a standard panel (Table 2). Except for the bee, *Hypotrigona* sp. (Hymenoptera), both expectations were met for all taxa, with a control to standard mean density ratio of 0–0.003 (Table 2). This low ratio suggests that the high-altitude flight of most insects was reduced during the crepuscular periods, during which the panels were launched and retrieved, compared with night period. For *Hypotrigona* sp., however, the ratio of mean control to standard panel was 0.167, suggesting that ~ 17% of its aerial density could have been collected near the ground. For this reason, this taxon was removed from further analysis.

**Table 2:** Overall abundance and occurrence of selected taxa in aerial samples collected on standard panels (220 panels between 40 and 290 m agl, in 125 sampling nights) and control panels (508 nets between 40 and 120 m agl).

	Standard panels (222 panels in 125 sampling nights)									Control panels (508 panels)							
Taxon	Mean panel density	Lower 95% CI^a^	Median panel density	Panel Freq^b^	Night Freq^c^	Total	Max panel density	Var / mean	Mean control/ standard^d^	Mean panel density	Upper 95% CI ^e^	Four min Standard^f^	Med panel density	Panel Freq^b^	Total	Max panel density	Var/ mean
*Dysdercus* sp.	0.03	− 0.007	0	0.02	0.04	7	4	2.7	0.0000	0	nd	0.0001	0	0.0000	0	0	nd
*Cy. endeca*	5.00	3.9325	1	0.54	0.67	1110	44	13.0	0.0016	0.0079	0.0156	0.0230	0	0.0079	4	1	0.993
*M. nitidus*	1.99	0.7636	0	0.27	0.43	442	117	43.2	0.0010	0.002	0.0058	0.0092	0	0.0020	1	1	1
*N. modulatus*	0.40	0.1898	0	0.12	0.21	88	17	6.2	0.0000	0	nd	0.0018	0	0.0000	0	0	nd
*A. coluzzii*	0.09	0.0519	0	0.09	0.17	21	2	1.1	0.0000	0	nd	0.0004	0	0.0000	0	0	nd
*P. sabaeus*	2.66	1.2729	0	0.29	0.42	591	134	41.4	0.0000	0	nd	0.0122	0	0.0000	0	0	nd
*P. fuscipes*	0.38	0.0414	0	0.09	0.21	84	32	17.2	0.0000	0	nd	0.0017	0	0.0000	0	0	nd
*Z. rhytidera*	8.61	5.0629	1	0.53	0.61	1912	246	83.6	0.0000	0	nd	0.0396	0	0.0000	0	0	nd
*Ch. coletta*	11.31	8.1055	2	0.58	0.71	2511	174	51.9	0.0009	0.0099	0.0185	0.0520	0	0.0099	5	1	0.991
*Hydrovatus* sp.	0.63	0.3414	0	0.18	0.34	139	21	7.4	0.0000	0	nd	0.0029	0	0.0000	0	0	nd
*Berosus* sp.	0.67	0.3805	0	0.19	0.32	149	20	7.2	0.0030	0.002	0.0058	0.0031	0	0.0020	1	1	1
*Microchelonus* sp.	0.26	0.1132	0	0.10	0.19	57	11	4.6	0.0000	0	nd	0.0012	0	0.0000	0	0	nd
*Hypotrigona* sp.	0.19	0.0519	0	0.05	0.08	42	8	5.7	0.1670	0.0316	0.0754	0.0009	0	0.0316	16	11	7.982
Overall	2.48			0.24	0.34	7153		21.9	0.0005	0.0018				0.0018	27		0.996

^a^ The lower 95% confidence interval (CI) of the mean panel density of standard panel used to compare with the upper 95% CI of the control panel for each taxon (see text).

^b^ Panel Frequency – Frequency of panels with at least one specimen per taxon.

^c^ Night Frequency – Frequency of nights with at least one specimen per taxon per night regardless of village (ie., includes nights when launches occurred in more than one village, n = 96).

^d^ Overall mean ratio of control/standard panel and mean control panel density were computed excluding *Hypotrigona* sp. (see text).

^e^ Expected density assuming insects were intercepted while the control panels reached over 20 m and remain there for ~ four minutes (see text).

### Overall abundance, sampling distribution, and correlation between taxa

**Figure 2:**
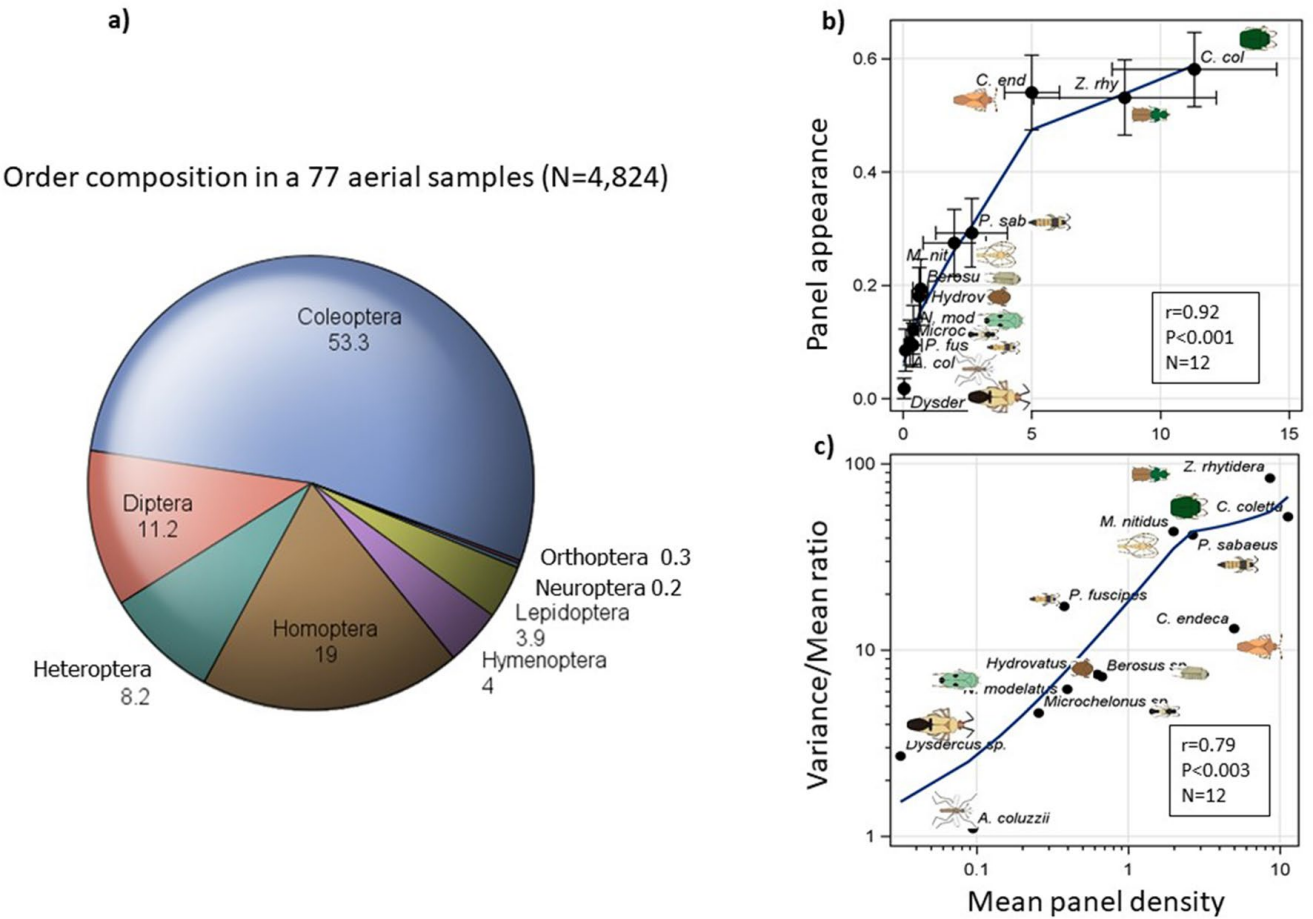
(**a**) Overall diversity (by insect orders) of aerial collection estimated based on samples from 70 sticky nets. Orders represented by less than 3 specimens (Blattodea, Thysanoptera, Megaloptera, Psocoptera, and Phasmatodea) are not shown. (**b**) Relationship between overall species density/panel (+ 95% CI) and the fraction of nets on which capture occurred on (+ 95% CI) as a measure of the regularity of high altitude flight activity. Insets show the Pearson correlation coefficient (r), its P value (P) and sample size (N). Schematic insect silhouettes are not to scale. (** c**) The relationship between the variance to mean ratio and its mean panel density.

Mean panel density of the selected taxa ranged over two orders of magnitude, from 0.05 for the *Dysdercus* sp. (Hemiptera) and *Anopheles coluzzii* (Diptera) to 11 for *Chaetocnema coletta* (Coleoptera, Table 2, Fig. 2b). The distribution of captured insects on (standard) panels was “L shaped,” typical of clumped distributions (Fig. S2), with a median panel density of zero for all species except *Ch. coletta*, *Cysteochila endeca* (Hemiptera) and *Zolotarevskyella rhytidera* (Coleoptera, Table 2), and a maximum of 246 specimens per panel, suggesting that flight activity was concentrated on one or a few nights. The frequency of nights with at least one specimen per taxon per night (nightly occurrence frequency) varied between 4 and 71% (Table 2), indicating that all taxa engaged in high-altitude flight activity over multiple nights. Moreover, panel occurrence frequency was positively correlated with taxon panel density (r_P_ = 0.92, P < 0.001, N = 12, Fig. 2b), indicating that taxa that appeared on fewer nights were the least abundant. Nonetheless, the high values of the variance to mean ratios (2.7–83.6, Table 2 and Fig. 2c) of all taxa, except *A. coluzzii*, suggest that the distribution of insects was temporally clustered.

**Figure 3:**
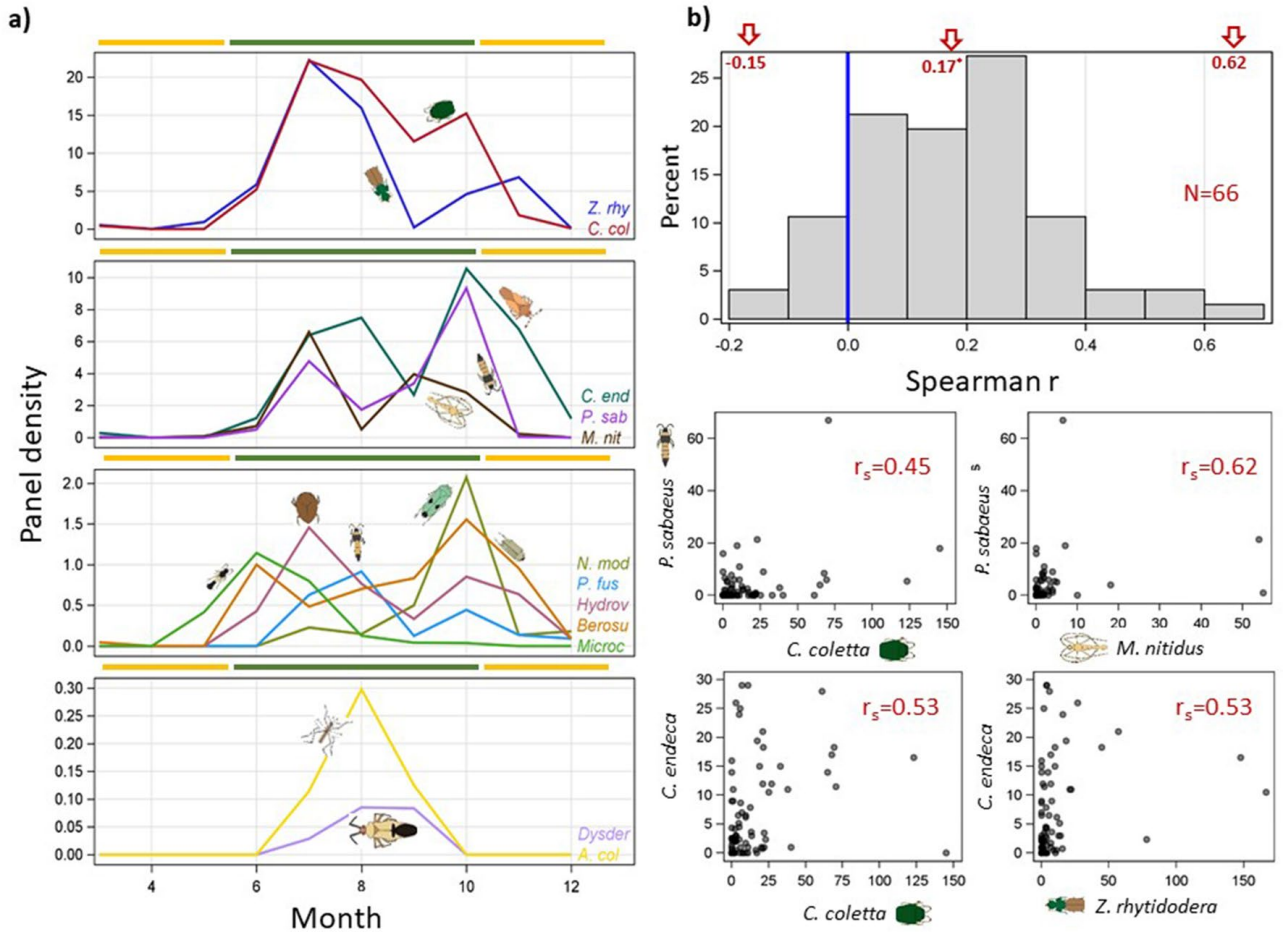
Temporal variation in flight activity across taxa. (**a**) Seasonal variation of migrant insects measured by panel density based on three-year data. Dry and rainy season are shown by yellow and green colors (ruler). (**b**) The distribution of the Spearman correlation coefficient (r_s_) between 66 pairs of migrant insects and relationship in nightly mean densities of the taxa pairs with highest Spearman correlation coefficients (b, N = 96 nights). Schematic insect silhouettes are not to scale (species names are truncated to conserve space).

All taxa exhibited marked seasonality in high-altitude flight activity (Fig. 3a), peaking between July and October, following considerably lower activity in May–June. Overall, flight declined substantially in November–December, and virtually none was recorded in March–April. Visual examination of the seasonality of individual taxa (Fig. 3a) suggests variability among species in flight activity. For example, *Microchelonus* sp. (Hymenoptera) appeared as early as May and peaked in June, whereas, the leafhopper, *Nephotettix modulatus* (Hemiptera) first appeared in July and peaked in October (Fig. 3a). A unimodal activity best describes *A. coluzzii*, *Dysdercus* sp., *Microchelonus* sp., and *N. modulatus*, while bimodal activity describes the other taxa, e.g., *Paederus fuscipes* (Coleoptera), and *Berosus* sp. (Coleoptera, Fig. 3a). Bimodal distribution was also suggested by the total-insect density/panel (Fig. S3). Likewise, correlations between taxa in nightly flight were low (Spearman, r_S_ mean = 0.17, − 0.15 < r_s_ < 0.62, n = 96, Fig. 3b). The highest r -values involved high density taxa, e.g., *Paederus sabaeus* and *Metacanthus nitidus* (r_s_ = 0.62, n = 96, P < 0.001). The mean pairwise correlation dropped to 0.09 (− 0.23 < r_s_ < 0.56, n = 77, Fig. 3b) after confining the correlation to the migration season (excluding the dry season, when migration was negligible).

### Variation between collection sites, years and altitude

**Figure 4:**
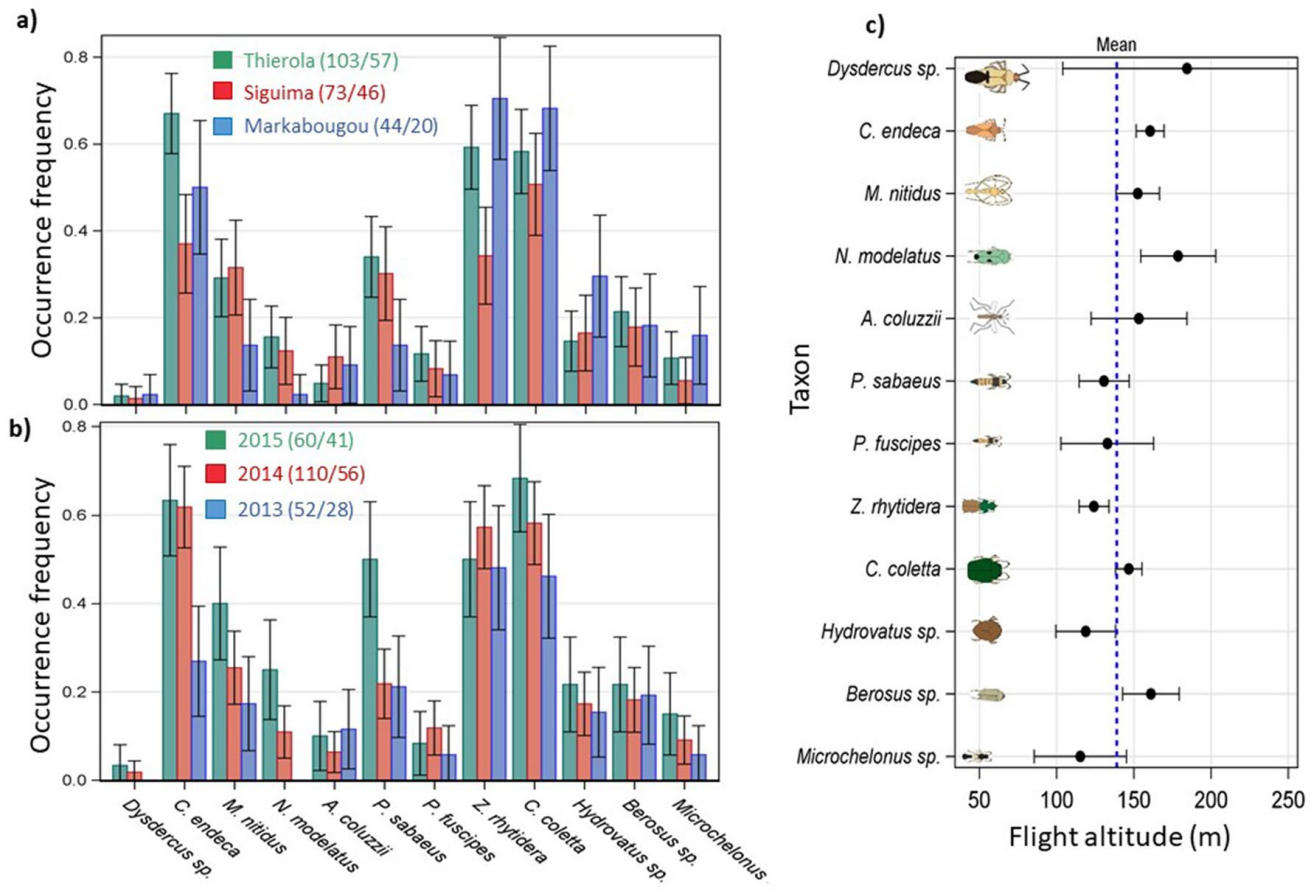
Spatial and annual variation in high altitude migration. Mean frequency of occurrence (+ 95% CI) of each taxon per panel by (**a**) locality (excluding Dallowere which was sampled in only 2 nights) and (**b**) year. The sampling effort in each year with respect to nets and nights is given in the legend. Between-species variation in flight altitude measured as mean panel altitude (+ 95% CI) weighted by panel density (**c**). Dotted blue line shows mean panel altitude. Note: the highest panel was typically 190 m agl, but between August and September 2015 we used a larger helium balloon and the highest panel was set at 290 m agl (see Methods). Schematic insect silhouettes are not to scale.

The occurrence frequency of each taxon was compared between localities (up to 100 km apart) and years to assess if they were location- or year-specific (Fig. 4). All taxa were found in all locations. The similarity between the localities in the appearance of each taxon is striking since they were partly sampled in different months (Fig. 1). Similarly, all taxa were present in every sampling year except for *Dysdercus* sp. and *N. modulatus*, which were not sampled in 2013. The rarity of *Dysdercus* sp. may account for its absence from the sparse data in 2013, which consisted of 28 sampling nights vs. 41 and 56 in the other years. Likewise, *N. modulatus* appeared late in the season (peaks in October) during 2014 and 2015, thus, it was unlikely to be sampled in 2013, in which collection ended by mid-August.

The typical flight altitude, measured as an average panel height weighted by the taxon’s panel density values, varied among taxa from 130 m (*Microchelonus* sp.) to 175 m (*N. modulatus*, Fig. 4c). Despite limited use of the largest balloon (3.3 m in diameter), which allowed sampling up to 290 m agl in Thierola between August–September 2015, all taxa were collected in the top panels (240–290 m agl).

### Aerial density and the effects of weather on high-altitude migration

Estimated aerial density of each taxon, (per 10^6^ m^3^ of air, Methods), was positively correlated with panel density (r = 0.93, P < 0.001, Fig. S3: Inset), suggesting that the results of analyses based on panel- and aerial- density would be similar. This correlation stemmed in part from the modest variability in average nightly wind speed at flight altitude (mean = 5.1 m/s, and the 10^th^ and 90^th^ percentiles are 2.4 and 8.0 m/s, respectively, Fig. S4).

Similar to the results based on panel density (Fig. 4), variation due to year and locality (village) of sampling were not significant in all taxa (P > 0.05, Table 3), whilst seasonality was significant in seven taxa (P < 0.05, Table 3 and Fig. 3a), and a modest effect of altitude was detected in four taxa (P < 0.05, Table 3). In subsequent models, the factors locality and year were therefore removed.

**Table 3:** Variation in taxon’s aerial density among years, locality (villages), altitude, and meteorological conditions (GLIMMIX models of random (year and village) and fixed (season, panel height, wind speed and direction, temperature and RH at flight height, 222 nets between 40 and 290 m agl, in 125 sampling nights).

Model (GLIMMIX)	Parameter	Dysdercus sp.	Cy. endeca	M. nitidus	N. Modulatus	A. coluzzii	P. sabeus	P. fuscipes	Z. rhytidera	Ch. coletta	Hydrovatus sp.	Berosus sp.	Microchelonus sp.
None	Var/Mean (mean)	3.5 (0.05)	27.3 (8.1)	108.8 (3.8)	12.3 (0.74)	2.3 (0.17)	59.3 (4.6)	14.5 (0.45)	142.9 (13.5)	116.9 (20.6)	16.8 (1.3)	17.0 (1.4)	9.6 (0.40)
Random vars: *Poisson*	Pearson χ^2^/df (BIC)	2.65 (111)	24.26 (4155)	57.55 (3925)	5.98 (737)	2.30 (245)	34.19 (3654)	8.42 (617)	59.75 (7617)	91.78 (11,867)	10.81 (1216)	16.07 (1498)	5.19 (552)
Random vars: Neg. Bin	Pearson χ^2^/df (BIC)	0.5 (59.2)	0.7 (1199)	1.4 (663)	0.5 (309)	0.7 (188)	0.8 (751)	1.1 (240)	0.9 (1241)	0.9 (1450)	0.6 (441)	0.8 (464)	0.9 (2344)
	Scale^a^	103.2^ ns^	4.1^ne^	10.4***	13.4***	12.9**	10.2^ne^	24.7^ne^	4.8***	5.0^ne^	14.1***	13.9***	21.3**
Fixed & random vars:	Pearson χ2/df (BIC)	0.3 (57.4)	1.0 (1131)	1.0 (626)	0.9 (286)	0.4 (162)	0.8 (699)	0.6 (227)	1.0 (1198)	1.4 (1386)	0.8 (426)	0.8* (463)	0.7 (229)
Negative Binomial	Scale^a^	30.7^ ns^	2.5ne	6.8***	9.2***	4.7**	5.5*	13.9^ne^	3.8***	3.3***	10.2***	12.1**	11.3**
	Year^b^ (SD)	0 (ne)	0.0^ne^ (ne)	0.001^ ns^ (0.13)	0^ne^ (ne)	0^ne^ (ne)	0.43^ne^ (0)	0^ne^ (0)	0^ne^ (ne)	0.09^ ns^ (0.13)	0^ne^ (ne)	0^ne^ (ne)	0^ne^ (ne)
	Village^b^ (SD)	0 (ne)	0.2ne (0)	0^ne^ (ne)	0^ne^ (ne)	0ne (ne)	0 (ne)	0.17ne (0)	0^ne^ (ne)	0.10^ ns^ (0.15)	0^ne^ (ne)	0^ne^ (ne)	0^ne^ (ne)
	Period^c^	Sep^ns^	October^ns^	Sep***	Oct**	Aug^ns^	Sep***	Aug***	July***	July***	July**	October^ns^	July^ns^
	Panel Height	0.02^ ns^ (0.022)	0.007*** (0.002)	0.002^ ns^ (0.004)	0.010*** (0)	0.003*** (0)	− 0.005*** (0)	− 0.016*** (0)	− 0.004^ ns^ (0.003)	0.007^ ns^ (0.004)	− 0.01* (0.006)	− 0.004^ ns^ (0.005)	− 0.004*** (0)
Fixed variables:	Pearson χ^2^/df, (BIC)	0.28 (60.8)	1.2 (1135)	1.2 (618)	0.5 (290)	0.3 (162)	2.3 (683)	0.4 (223)	0.9 (1202)	2.0 (1357)	0.6 (421)	0.9 (450)	0.8(228)
Negative Binomial	Scale^a^	41.2^ ns^	2.7***	6.8***	8.4***	4.1**	4.^7^***	12.4***	3.6***	2.8***	9.6***	9.8***	11.8***
	Period^d^	Jul-Sep^ns^	Oct-Dec***	Oct-Dec**	Oct-Dec^ns^	Jul-Sep^ns^	Oct-Dec^ns^	Oct-Dec^ns^	Jul-Sep**	Jul-Sep*	Oct-Dec^ns^	Oct-Dec^ns^	Mar-Jun^ns^
	Wind dir. vector (N-S)^e^	1.99^ ns^	− 0.59^ ns^	0.6^ ns^	− 0.19^ ns^	0.12^ ns^	0.09^ ns^	− 1.07 ns	− 0.82^ ns^	− 0.75^ ns^	0.13^ ns^	0.22^ ns^	0.12^ ns^
	Per x Wind dir. (N-S)^f^	1.89^ ns^	− 0.30^ ns^	0.96^ ns^	− 0.39^ ns^	0.02^ ns^	− 0.56^ ns^	− 3.0^ ns^	0.01^ ns^	0.18^ ns^	0.40^ ns^	− 0.10^ ns^	− 0.80^ ns^
	Wind speed ^g^	− 0.23^ ns^	− 0.12^ ns^	− 0.37**	− 0.41*	− 0.17^ ns^	− 0.46***	0.43*	− 0.09^ ns^	− 0.26***	− 0.44***	− 0.41***	− 0.80^ ns^
	Temperature (^o^C )^g^	1.04^ ns^	− 0.06^ ns^	0.81**	− 0.02^ ns^	− 0.31^ ns^	0.72**	0.30^ ns^	− 0.10^ ns^	0.09^ ns^	− 0.06^ ns^	0.08^ ns^	0.94*
	RH (%)^g^	0.23 ns	0.01 ns	0.11***	0.03^ ns^	− 0.001 ns	0.17***	0.05^ ns^	− 0.01^ ns^	0.04 **	0.03^ ns^	0.03^ ns^	0.20 *

^a^For negative bionomial scale parameter estimates the k parameter of this distribution.

^b^The effects of year and village could not be estimated simultaneously, so the estimates were produced in two separate models, each including only one of the factors.

^c^Two month periods were used (Mar-Apr, May-Jun, Jul-Aug, Sep-Oct, and Nov-Dec). The period of highest panel density is shown with its statistical significance.

***^,^**^,^*^, ns, ne^ refer to significance probability of 0.001, 0.01 and 0.05, > 0.05, and to parameters that could not be estimated, respectively.

**Figure 5:**
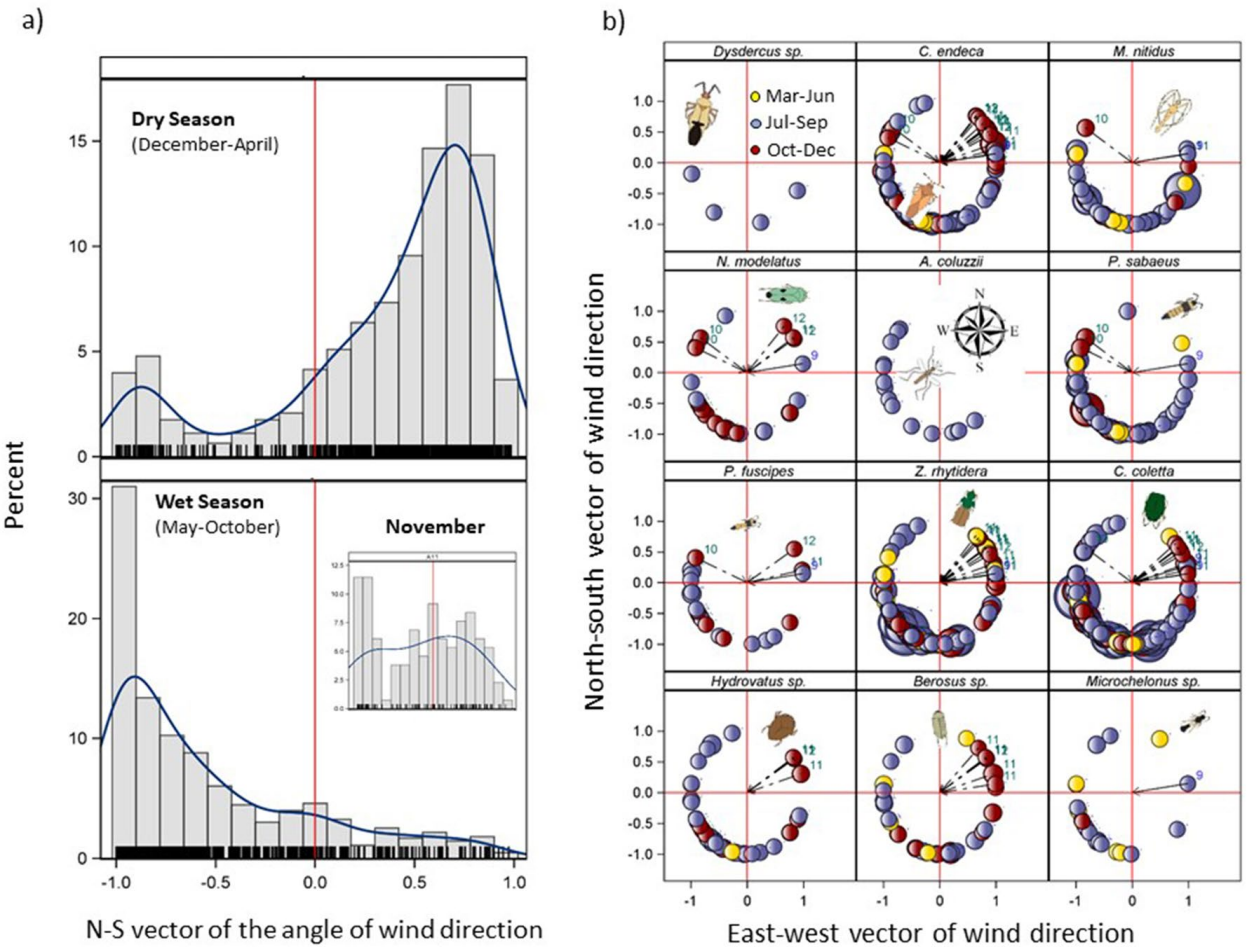
Seasonality of the south-north component of nightly wind direction in the Sahel and nightly wind direction during high-altitude flights of each taxon. (**a**) To explore the possibility of north–south migration into the Sahel from more equatorial regions, the north–south component of nightly wind direction (2012–2015 MERRA2 data; all nights) shows the frequencies of winds during the dry (top) and wet (bottom) season in Thierola (the other villages exhibited similar distributions). Kernel distributions are shown in blue. Wind direction from the N and S are indicated by positive and negative south–north vector values, respectively. INSET: November is a transition month with variable wind direction. Red reference line at the origin indicates easterly or westerly winds. Fringe marks indicate actual values south-north component of wind direction. (**b**) Wind direction during high-altitude flights of selected taxa. Circles denote source of mean nightly winds in relation to the capture location (origin) with north and east denoted by top and right red lines, respectively. Circle size reflects nightly aerial density and their color denotes the period (top left). Dotted arrows highlight southbound winds during the end of the wet season, that could be used for the “return” migration from the Sahel towards tropical areas closer to the Equator (numbers denote the months of such events). Schematic insect silhouettes are not to scale.

^d^To better reflect seasonal variation in wind direction (Fig. 5a), periods for this analysis were Mar-Jun, Jul-Sep, and Oct-Dec. The period of highest panel density is shown with its statistical significance.

^e^The main effect of wind direction, measured from south (− 1) to north (1) of each sampling location on aerial density. This measures the south-north component of the average angle of nightly wind direction (see text and Fig. 5a).

^f^The interaction between Period and S–N vector of wind is shown for the difference between Oct-Dec and Jul-Sep.

^g^Measured at flight altitude using MERRA2 database based on panel height (see text for details).

To assess if migration activity occurred during particular weather conditions near the ground or at flight-height, we compared the taxon’s mean temperature weighted by its aerial density with that of other taxa and similarly considered the relative humidity (RH), and wind speed (Fig. S5). Variation across taxa was moderate and sizable overlap was apparent among their 95% CI (Fig. S5). Flight activity for most taxa occurred across broad temperature ranges and their 95% CI intersected the wet season mean temperatures at the ground (8 taxa) and at flight altitude (9 taxa, Fig. S5a). Overlapping CI of most taxa were also common with RH and wind speed, although most insect flight took place at lower RH and lower wind speed than their wet season averages (Fig. S5b and S5c), possibly because rainstorm conditions inhibit migration^24^ or because aerial sampling did not include such nights. Statistical models that evaluated the effects of these weather parameters on high-altitude activity revealed that flights of certain taxa were more common under lower wind speed (seven taxa) and higher RH (four taxa, Table 3).

### Seasonal wind and flight directions

As expected^31^, wind direction in the Sahel showed marked seasonality, with northerly winds (blowing towards the south) dominating from December to April and reversing course from May to October (Fig. 5a). However, in November, winds are variable and blow towards the south and the north with similar frequencies (Fig. 5a).

All taxa exhibited frequent northward migrations on southerly winds, which ranged widely from WSW to the ESE during the wet season (Fig. 5b). During the wet season (July–September), Sahelian rainfall is associated with large mesoscale convective systems with squall lines which have changeable wind directions. So, insects could have been carried by winds to nearly all directions (Fig. 5) and dispersed widely across the Sahel, albeit with different intensities. The concentration of the circles, denoting the source of mean winds, and their size, which correspond to aerial density (Fig. 5b), signifies the relative position of the source populations. For example, *Z. rhytidera* exhibited a strong influx from the southwest, and *Ch. coletta* from western and southern sources.

To assess if a movement back into the savannas south of the Sahel took place at the end of the rainy season, we examined whether insects exhibited movement with southbound winds during September to December (Fig. 5b). Although they were less common, at least one or a few nights’ migration on southward wind were recorded in all except the three least abundant taxa (*Dysdercus* sp., *A. coluzzii*, and *Microchelonus* sp., Fig. 2c). Such southward movements were especially frequent in *Z. rhytidera*, *Cy. endeca*, *Ch. coletta*, and *Berosus* sp. (Fig. 5b). Tests of wind “selectivity”, evaluating if aerial density was higher during nights with favorable wind direction (southward during the end of the wet season) were performed using contingency tables contrasting the proportion of nights with northbound and southbound flights during October through December did not support selective southward flight across taxa (P > 0.05 at the individual test level, not shown). Similarly, no significant interaction of wind direction by period was detected in the aerial density analysis (Table 3).

## Discussion

This study presents the first cross-season survey of high-altitude migrant insects in Africa. Based on 125 high-altitude sampling nights, yielding 222 samples, we assessed the diversity of migrants and focusing on a dozen taxa, evaluated their compositional regularity, aerial abundance, movement direction, and relationships with key meteorological conditions. This information is fundamental to understanding the scope and impacts of African insect migration and can inform on the value of monitoring aerial migration to address African food security, public health, and conservation issues.

The composition of our collection (Coleoptera—53%, Hemiptera—27%; especially *Auchenorrhyncha*, and Diptera—11%, Table 1) was distinct from aerial collections in Europe, which was dominated by Hemiptera (especially aphids) and Hymenoptera^32^, and in North America^5^, which was dominated by Diptera and Coleoptera, possibly reflecting taxa more tolerant of xeric environments. The large number of taxa representing a hundred families from thirteen orders already identified from a small fraction of the aerial collection (< 10%, Tables 1, and S1) suggests that migration at altitude is a common and widespread life history strategy in the Sahel, as expected from the impermanence of many habitats^33,34^. Although our taxa selection depended on ease of identification and repeatable appearance in the first subsamples evaluated, we later realized that four represent notable agricultural pests: *Dysdercus* sp., *N. modulatus, Ch. coletta*, and *Cy. endeca;* three affect public health: *P. sabaeus* and *P. fuscipes*, which cause outbreaks of severe dermatitis^35^ and the African malaria mosquito, *A. coluzzii*, and six are predators that likely control pests and mosquitoes (e.g., *Microchelonus* sp. and *Hydrovatus* sp. (Dytiscidae), Table S2). Moreover, our results explain the outbreaks of dermatitis due to *Paederus* beetles in Africa^36^. The services provided by an arbitrary assortment of windborne migrants suggests that further studies of insect migration including aerial surveillance may provide useful insights into the causes of human, animal, and plant disease outbreaks. Among the insect genera identified, several included known or suspected windborne migrants: *Dysdercus* sp.^37^, *A. coluzzii*^15,38–40^, and *M. nitidus*^41^; for the rest, such knowledge is new, as is their reported presence in Mali. Clearly, this aerial collection awaits additional study. Insects flying > 200 m agl and day-flyers were underrepresented in our collection, as were larger insects (e.g., grasshoppers, moths) that could detach themselves from the thin layer of glue. Also, tiny insects (e.g., aphids and midges) might have been overlooked when insects were manually extracted from the panels.

Migration regularity was demonstrated by the similarity of the taxa composition over multiple years (2013–2015), in locations up to 100 km apart, and seasonal highs during the wet seasons (Figs. 3,4 and Table 3)suggesting that it is integral behavior in these taxa. The hypothesis that migration occurred exclusively on particular nights, was rejected because the nightly occurrence frequencies varied between 4 and 71% and because the positive correlation between the taxon’s overall abundance and the nightly occurrence frequency (Fig. 2), indicated that low nightly occurrence reflected the low overall taxon abundance. The low inter-species correlations in nightly densities indicated species-specific migration patterns rather than rare mass-migration events. Altogether, the results suggest that these taxa engaged in migration over many nights, rather than during a few rare events; albeit certain nights, probably near peak activity were of higher density (Figs. 3a, S3). Indeed, accommodating variation due to season, year, village, and altitude, models with a negative binomial error distribution were superior to those with *Poisson* distributions in all taxa (Table 3), suggesting that migration fluctuated during the wet season. The cross-panel occurrence (Table 2) indicated that clumping does not reflect tight flying “swarms”.

**Figure 6:**
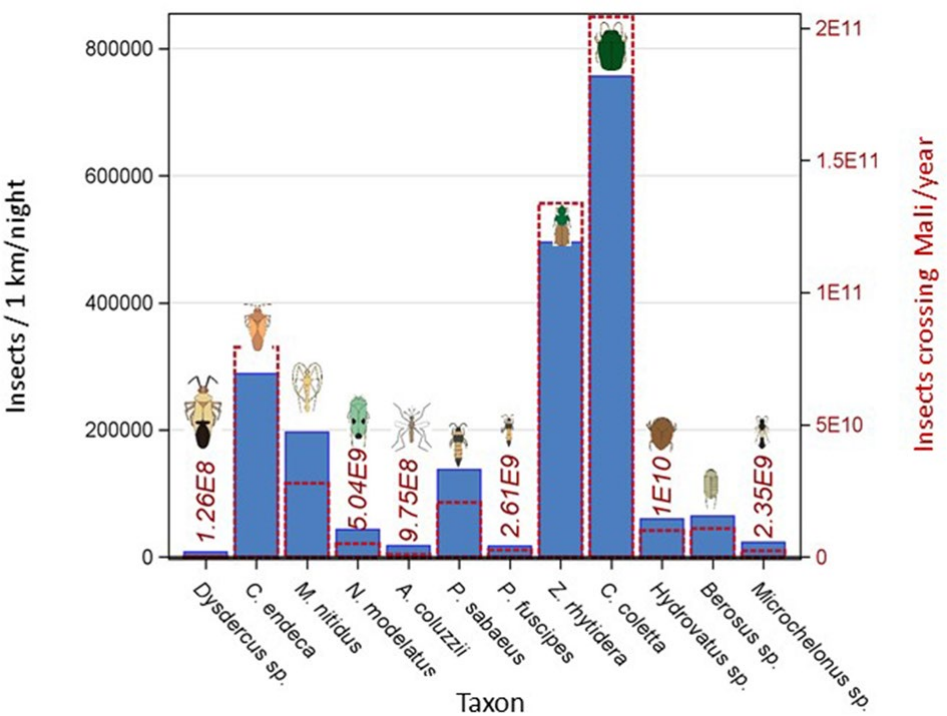
The number of insects per species crossing at altitude (50–250 m agl) imaginary lines perpendicular to the prevailing wind. Migrants per night per 1 km (left Y axis, blue) are superimposed on the annual number per 1,000 km line across Mali (right Y axis, red, see text).

The magnitude of migration is illustrated as the number of insects expected to cross a 1 km line perpendicular to the wind at altitude over a single night. Because our taxa were captured in altitudes spanning 40 to 290 m agl, a conservative estimate of the depth of the flight layer is 200 m. Using the average nightly wind speed (3.5 m/s, Fig. S5c), we estimated the number of insects crossing this imaginary line throughout the night (14 h sampling duration), yielding an average parcel of air of 176.4 km length, 1 km (width), and 0.2 km (height). The average aerial density was calculated across all sampling nights (including zeros) during the species’ “migration period”, estimated as the longest annual interval when migration occurred (between the first and the last dates the taxon was captured). The number of insects per taxa crossing the 1 km line each night, between 50 to 250 m agl ranged from 7800 (*Dysdercus* sp.) to 750,000 (*Ch. coletta*, Fig. 6). Extrapolating these values to the annual number of insects crossing the 1000 km line spanning Mali’s width at latitude 14.0°N suggests values between one hundred million (*Dysdercus* sp.) and 0.1 quadrillion (10^14^ *Ch. coletta*). The mean total insect density/panel (280, Fig. S3) is > 25 times greater than that of *Ch. coletta*, our most abundant species (Table 2). Considering that these conservative values represent nocturnal migration of single taxa over mere 200 m layer in depth, they underscore the enormous scale of these movements and dwarf the number of insects flying above the UK, which, when converted from the observed 300 km line to 1000 km would total 10^13^ (for all insects)^22^.

Flight speeds of small insects (< 3 mg; Table S2) range around 1 m/s^42,43^, thus their overall displacement at altitude, where typical wind speed exceeds 4 m/s (Fig. S5, S3, and below) is governed by the wind. The distance covered by windborne migrants depends on wind speed and the duration of their flights. Using a conservative average of wind speed at altitude of 4 m/s (Fig. S5 and S3), an insect flying, for 2–10 h would be transported 30–140 km, respectively. Flight durations can be estimated by flight mills^40,44,45^ or, less often, by the distance that they demonstrably flew and knowledge of the wind speed. Flight mill data suggest that leafhoppers (*N. virescens*) can fly over 10.75h^46^, similar to *A. gambiae* s.l. (10 h)^47,48^. Because all taxa were collected in the top panels, our sampling has not reached their highest flight altitude and our results likely underestimate the actual flight altitude as well as total abundance, diversity, speed and displacement distance. Indeed, radar data have shown that migrants reach (and often exceed) 450 m agl^24,49–51^. The low abundance of *Dysdercus* sp. may be accounted for by incomplete altitude sampling, as is the case for *N. modulates,* which tend to fly at higher altitudes (Fig. 4c). This variation between species suggest that low-flying insects including *P. sabaeus*, *Z. rhytidera*, and *Hydrovatus* sp. may engage in shorter flights than high-flying taxa, e.g., *N. modulatus*, *Cy. endeca*, *Berosus* sp., and *M. nitidus*.

Meteorological radar data from the Sahel revealed wind speed means between 8 and 12 m/s, with occasional nights when wind speed in the lower jet stream (LJS, 150–500 m agl) exceed 15 m/s^52^. Reanalysis datasets such as MERRA-2 or ERA5 consistently underestimate wind speed in that layer^53^. Because migrant insects often concentrate at the layer with maximal air speed^24^, a small insect flying between 1 and 10 h in a realistic average windspeed of the LJS (10 m/s) will cover on average 36–360 km per night (over 500 km in some nights).

The seasonally productive habitats of the Sahel border diverse and “teeming” sub-equatorial habitats; a combination that may increase the abundance of insect migrants and account for concentration of aerial predators such as swifts^54,55^, nightjars^56^, and bats^57^ during peak migration in this region. During their fall migration, swifts arrive in the Sahel (latitudes 11–15°) in mid to late August when insect migration peaks (Fig. S3), and remain in the area for ~ 24d, 30% (9–67%) of their total migration duration, whilst covering only 9% of their total route. This contrasts with their spring migration in May, before insect migration builds up, when they stay in the Sahel ~ 4d, constituting only 14% (3–38%) of their journey^55^. It appears that the swifts rely on the extreme insect abundance before heading to equatorial regions, where they overwinter, suggesting it is greater than in equatorial regions. Hence, it may represent a global hot zone for migratory insects.

The seasonal movement of the Inter-Tropical Convergence Zone (ITCZ), which marks the zone of precipitation, implies continuously shifting resources across the girth of the Sahel . During its short wet season a mosaic of patches receive high and low rainfall in any given year, which in turn reinforces migration^11,33,34,58,59^. Movement between resource patches is predicted, especially for inhabitants of ephemeral water such as puddles, e.g., *A. coluzzii*. Marked seasonality with migration peaking during the rainy season was evident in most taxa and might have been found in all taxa, had larger sample sizes been available (Table 3 and Fig. 3). Migration dynamics in nine taxa exhibited bimodal activity similar to the total density of insects/panel (Fig. S3); only *A. coluzzii*, *Dysdercus* sp., and *Microchelonus* sp. exhibited a unimodal pattern (Fig. 3). A wide unimodal migration peak fits the “residential Sahelian migration” strategy in species that persist in the Sahel throughout the year, but continuously migrate into new environments to maximize exploitation of resource-rich patches and safeguard against severe wet-season droughts^31^ that could eliminate local populations. This strategy implies an ability to withstand the Sahelian dry season via dormancy as is the case for *A. coluzzii*^38,60–62^. Bi-modal flight activity better fits a “round-trip migration strategy”, whereby insects arrive in the Sahel from “perennial” habitats closer to the equator during the early peak, and “return” southwards before the approaching dry season, e.g., *Dysdercus volkeri* in Ivory Coast and Mali^37^. For example, the grasshopper *Oedaleus senegalensis* flies southward from the northern Sahel on Harmattan winds and covers 300–400 km in a night’s migration, although its northward movements seem gradual^7,24,58^. The late wet-season peak (October) in total insect density/panel (Fig. S3) supports a rise in population density as predicted. However, contrary to prediction, wind direction during the seven nights with highest total insect density had a predominant northward component (not shown). These strategies are not mutually exclusive as species may exhibit both “round-trip migration” and “residential migration” in different populations. For example, equatorial populations of *A. coluzzii*^63–65^, are not expected to extensively engage in windborne migration. Likewise, O. senegalensis (Acrididae) probably uses both strategies. It exhibits aestivation—eggs can survive several years in dry soil—but it can also cross the Sahel into the Savanna and return (over its 3 annual generations)^4,7,24,28,33^. The relative importance of each strategy may vary among populations. Possibly, species employing the “round trip” strategy may incorporate movements similar to “residential Sahelian migration” during the wet season, to better exploit the shifting rains and then return southwards to habitats with perennial resources, as exemplified by *O. senegalensis* (above). Distinguishing among these possibilities and linking them to life history traits require additional information, which currently are unverified for most Sahelian taxa (Table S2).

Wind directions during the period of flight activity spanned well over 180° for all taxa (Fig. 5 and Table 3), suggesting that movement between resource patches in the Sahel is widespread. For example, the nearly uniform distribution over large sectors exhibited by *Hydrovatus* sp. and *Berosus* sp. indicate dispersal with only weak concentration of southerly origin, probably reflecting migration between aquatic habitats in multiple directions. During the rains, movements northwards and eastwards were especially common, following the ITCZ. After the long dry season, rain signifies high productivity, minimal competition, predation, and parasitism^33^. During the end of the wet season, movement southward was observed in 10 of the 12 taxa, however, there was no evidence of selective flight on southbound winds as was found over Europe^22,66^. The prevailing seasonal winds in the Sahel—southwest monsoon and northeast Harmattan—happen to take the migrants in seasonally appropriate directions > 70% of the nights (Fig. 5a), reducing the pressure for wind selectivity that was demonstrated in temperate zones, where selectivity may confer greater benefit. Return migrations were possibly missed given the fewer sampling nights during October–December and because during this time the LJS may have been higher than 190 m and most insects flew above our traps.

In conclusion, our results demonstrate that a multitude of Sahelian insects regularly engage in high-altitude windborne migration, covering hundreds of kilometers, in enormous densities during the rainy season. The implications of this for ecosystem stability, public health, and especially for food security are profound. The dynamics of the studied taxa suggest species-specific drivers. The dominant winds— southerly monsoon during the wet season and northerly Harmattan—during the dry season structure the sources and destinations, yet, all taxa exploited winds that transported them to various directions, indicating an intra-Sahelian patch interchange.

## Methods

### Study area

Aerial sampling stations were placed in four Sahelian villages (Fig. 1): Thierola (13.6586, − 7.2147) and Siguima (14.1676, − 7.2279; March 2013 to November 2015); Markabougou (13.9144, − 6.3438; June 2013 to June 2015), and Dallowere (13.6158, − 7.0369; July to November 2015). Unless otherwise indicated, Dallowere, situated 25 km from Thierola was excluded from the statistical analysis because it was represented by only two sampling nights.

No aerial samples were taken during January and February (Fig. 1). The study area has been described in detail previously^38,61,67–69^. Briefly, it is a rural area characterized by scattered villages, with traditional mud-brick houses, surrounded by fields, beyond which is a dry savanna, consisting of grasses, shrubs, and scattered trees Over 90% of the rains fall in the wet season (June—October, ~ 550 mm annually), forming puddles and ponds that usually dry by November. Rainfall during the dry season is negligible (0—30 mm, December—May).

### Aerial sampling and specimen processing

The aerial sampling methods have been described in detail previously in a study that focused on *Anopheles* mosquito species from the whole collection^15^. Briefly, insect sampling was conducted using sticky nets (panels) attached to the tethering line of 3 m diameter helium-filled balloons, with each balloon typically carrying three panels. Initially, panels were suspended at 40 m, 120 m, and 160 m agl, but from August 2013, after preliminary results showed higher panel densities at higher elevations, the typical altitude was 90 m, 120 m, and 190 m agl. When a larger balloon (3.3 m dia.) was deployed at Thierola (August–September 2015), two additional panels were added at 240 m and 290 m agl. Balloons were launched approximately one hour before sunset (~ 17:00) and retrieved one hour after sunrise (~ 07:30), the following morning. To control for insects trapped near the ground as the panels were raised and lowered, comparable control panels were raised up to 40 m agl and immediately retrieved during each balloon launch and retrieval operation. Between September and November 2014, the control panels were raised to 120 m agl. The control panels typically spent 5 min above 20 m when raised to 40 m, and up to 10 min when raised to 120 m. Following panel retrieval, inspection for insects was conducted in a dedicated clean area. Individual insects were removed from the nets with forceps, counted, and stored in labeled vials containing 80% ethanol.

### Taxon selection and identification

Using a dissecting microscope, insects were sorted by morphotype—an informal taxon assigned to specimens with similar morphology that are putative members of a single species— counted and recorded in a database. The remaining insects were sorted to order, counted, and recorded. Selected morphotypes were chosen based on their easily identifiable features and their repeated appearance in a preliminary examination of the collection. Later, a subset were identified by expert taxonomists who narrowed the identification down to species or genus and confirmed that the morphotype likely represents a single species (Tables S1 and S2). The thirteen taxa used in the present study are described in Table S2 and Fig. S1.

### Data analysis

During months when aerial sampling was carried out in one, two or three sites, we sampled four, three, or two dates of collections per site, respectively. The dates were spread more or less evenly through the sampling days of each month. From each sampling night, two panels were selected in sequential order (120 m, 160 m, 190 m…) and 1–4 vials of insect specimens, representing > 30% of the total insects collected (based on the count of total insects removed from the panel, above), were sorted and counted as described above. For example, if the total insects removed from a panel in the field were 660 and the first vial had 185 insects, which were sorted, we added a second vial with 155 insects. Because the sum of the insects sorted was 340, which is > 30% of the 660 (220), no additional vial was sorted. Subsampling of the collection for the analysis was carried out to represent variation between years, seasons, sites, and altitude. However, as typical for field studies in remote areas, logistical constraints resulted in sampling that was not perfectly balanced (Fig. 1). The ‘panel density’ of the selected taxa was computed as the product of the total number of insects collected on that panel and the fraction of specimens from each taxon in the subsample sorted. Thus, using the example above, if the count of the first taxon was 9 in a subsample of 340/660, then the panel density was estimated as the 9*(660/340) = 17.47, which was rounded to 18.

The Modern-Era Retrospective analysis for Research and Applications (*MERRA-2*^70^)—selected to represent observed nightly conditions (18:00 through 06:00)—were used to calculate nightly mean temperature, relative humidity (RH), wind speed and direction. Corresponding values were computed for 2, 50, 70, 200, 330 m agl for the nearest grid center (available in ~ 65km^2^ resolution) of each village: Siguima, Markabougou and Thierola (Dallowere, located 25 km south of Thierola was included in the same grid of Thierola). Hourly records were available up to 10 m, and 3-hourly records at altitude > 10 m. Conditions at panel height, e.g., mean nightly wind speed, were estimated based on the nearest available altitude. The altitudes of the sampling panels were generally well above the insects’ ‘flight boundary layer’—the lowest air layer where an insect’s self-propelled flight speed is greater than wind speed—so flight direction is governed primarily by wind direction^24,71,72^. Thus, flight direction was estimated from the average nightly wind direction during the night of capture at the location and panel height. The seasonal flight direction was estimated as the weighted average nightly wind direction at the flight altitude at which a taxon was captured, with the taxon aerial density at the panel used as a weight to compute the weighted circular mean angle for that taxon (see below).

Aerial insect density was estimated based on the taxon’s panel density (above) and total air volume that passed through that panel that night, i.e.:

$$\begin{aligned} {\text{Aerial}}\,{\text{density}} & = {\text{total}}\,{\text{insects}}\,{\text{per}}\,{\text{panel}}/{\text{volume}}\,{\text{of}}\,{\text{air}}\,{\text{sampled}},\,{\text{and}}\, {\text{Volume}}\,{\text{of}}\,{\text{air}}\,{\text{sampled}} \\ & = {\text{panel}}\,{\text{surface}}\,{\text{area}}*{\text{mean}}\,{\text{nightly}}\,{\text{wind}}\,{\text{speed}}*{\text{sampling}}\,{\text{duration}} \\ \end{aligned}$$

Insect sampling duration was calculated from balloon launch time until its retrieval time (typically 17:30 to 7:30; ~ 14 h). Based on panel altitude, wind speed was selected from the nearest layer (above) to calculate the nightly average for each panel. Analysis was carried out both on panel density, and aerial density, to ensure that all key aspects of the data are well represented. The calculation of aerial density assumed that air passed through the net with minimal attenuation and that the panel remained perpendicular to the wind direction throughout. These are reasonable assumptions because observations showed no flipping of the panels, the thin layer of glue did not block the holes of the net, and insects were always found only on one side of the panel. Under these assumptions (panel surface = 3m^2^, for 14 h) and nightly average wind speed from the MERRA-2 database, the volume of air that would pass through the nets when average nightly wind speed was 1 vs. 7 m/s is 151,200 and 1,058,400m^3^, respectively.

The variation in the aerial density measured by each panel due to the effects of season, altitude, and other factors was evaluated using mixed linear models with either Poisson or negative binomial error distributions implemented by proc GLIMMIX with a log link function^73^. These models accommodate the non-negative integer-counts and the combination of random and fixed effects. The lower Bayesian Information Criterion (BIC), the significance of the underlying factors, the ratio of the Pearson χ^2^ to the degrees of freedom and the significance of the scale parameter (estimating k of the negative binomial distribution) were used to choose between models. Mean nightly wind direction was computed based on the average values of north–south and east–west vectors of the hourly wind direction angle (unweighted by wind speed). The dispersion of individual angles around the mean was measured by the mean circular resultant length ‘r’ (range: 0 to 1), indicating tighter clustering around the mean by higher values. Rayleigh’s test of uniformity was used to test whether there was no mean direction, as when the angles form a uniform distribution over 360 degrees.

## Supplementary information

Supplementary Information 1.

## Data deposition

The dataset will be deposited in Dryad.
