# Supplementary material for: Diversity, dynamics, direction, and magnitude of high-altitude migrating insects in the Sahel
Source: Sci Rep. 2020 Nov 25;10:20523. doi: 10.1038/s41598-020-77196-7 (PMC7688652; doi:10.1038/s41598-020-77196-7)
Supplement: Supplementary file 1 — Supplementary Information 1. [file 41598_2020_77196_MOESM1_ESM.pdf]

Supplementary Materials:

Diversity, dynamics, direction, and magnitude of high-altitude migrating insects in the Sahel

Jenna Florio[a], Laura M. Verú[a], Adama Dao[b], Alpha S. Yaro[b], Moussa Diallo[b], Zana L. Sanogo[b], Djibril Samaké[b], Diana L. Huestis[a], Ousman Yossi[b], Elijah Talamas[c,d], M. Lourdes Chamorro[c], J. Howard Frank[e], Maurizio Biondi[f], Carsten Morkel[g], Charles Bartlett[h], Yvonne-Marie Linton[j], Ehud Strobach[i], Jason W. Chapman[k], Don R. Reynolds[l], Roy Faiman[a], Benjamin J. Krajacich[a], Corey S. Smith[m] and Tovi Lehmann*[a]

a Laboratory of Malaria and Vector Research, NIAID, NIH. Rockville, MD, USA
b Malaria Research and Training Center (MRTC) / Faculty of Medicine, Pharmacy and Odonto-stomatology, Bamako, Mali
c Systematic Entomology Laboratory - ARS, USDA c/o Smithsonian Institution, National Museum of Natural History, Washington, DC, USA
d Florida Department of Agriculture and Consumer Services, Division of Plant Industry, Gainesville, FL, USA
e Entomology and Nematology Department, University of Florida, Gainesville, FL, USA
f Department of Life, Health, and Environmental Sciences, University of L'Aquila, Italy
g Institute of Applied Entomology, Beverungen, Germany
h Department of Entomology and Wildlife Ecology, University of Delaware, Newark DE, USA
i Earth System Science Interdisciplinary Center, University of Maryland, College Park, MD, USA
j Walter Reed Biosystematics Unit, Smithsonian Institution Museum Support Center, Suitland, MD, USA and Department of Entomology, Smithsonian Institution, National Museum of Natural History, Washington, DC, USA
k Centre for Ecology and Conservation, and Environment and Sustainability Inst., University of Exeter, Penryn, Cornwall, UK and College of Plant Protection, Nanjing Agricultural University, Nanjing, P. R. China
l Natural Resources Institute, University of Greenwich, Chatham, Kent, ME4 4TB, UK, and Rothamsted Research, Harpenden, Hertfordshire AL5 2JQ, UK
m American Museum of Natural History, New York, NY, USA
* Corresponding author's email address: tlehmann@niaid.nih.gov

The Supplementary Materials include: Figures S1-S5, Tables S1 (List of identified insects from the project) and S2 (selected taxa: taxonomy and natural history).

Figure S1. Plates 1-15. 1: *Dysdercus* sp. (Pyrrhocoridae), 2: *Cysteochila endeca* Drake (Tingidae), 3: *Metacanthus nitidus* Štusák (Berytidae), 4-6: *Nephotettix modulatus* Melichar (Cicadellidae), 7: *Anopheles coluzzii* Coetzee & Wilkerson (male, Culicidae), 8: *Paederus sabaeus* Erichson, 9: *Paederus fuscipes* Curtis (Staphylinidae), 10: *Zolotarevskyella rhytidera* (Chaudoir) (Carabidae), 11: *Chaetocnema coletta* Bechyn (Chrysomelidae), 12: *Hydrovatus* sp. (Dytiscidae), 13: *Berosus* sp. (Hydrophilidae), 14: *Microchelonus* sp. (Braconidae), 15: *Hypotrigona* sp. (Apidae). Ruler units are mm.

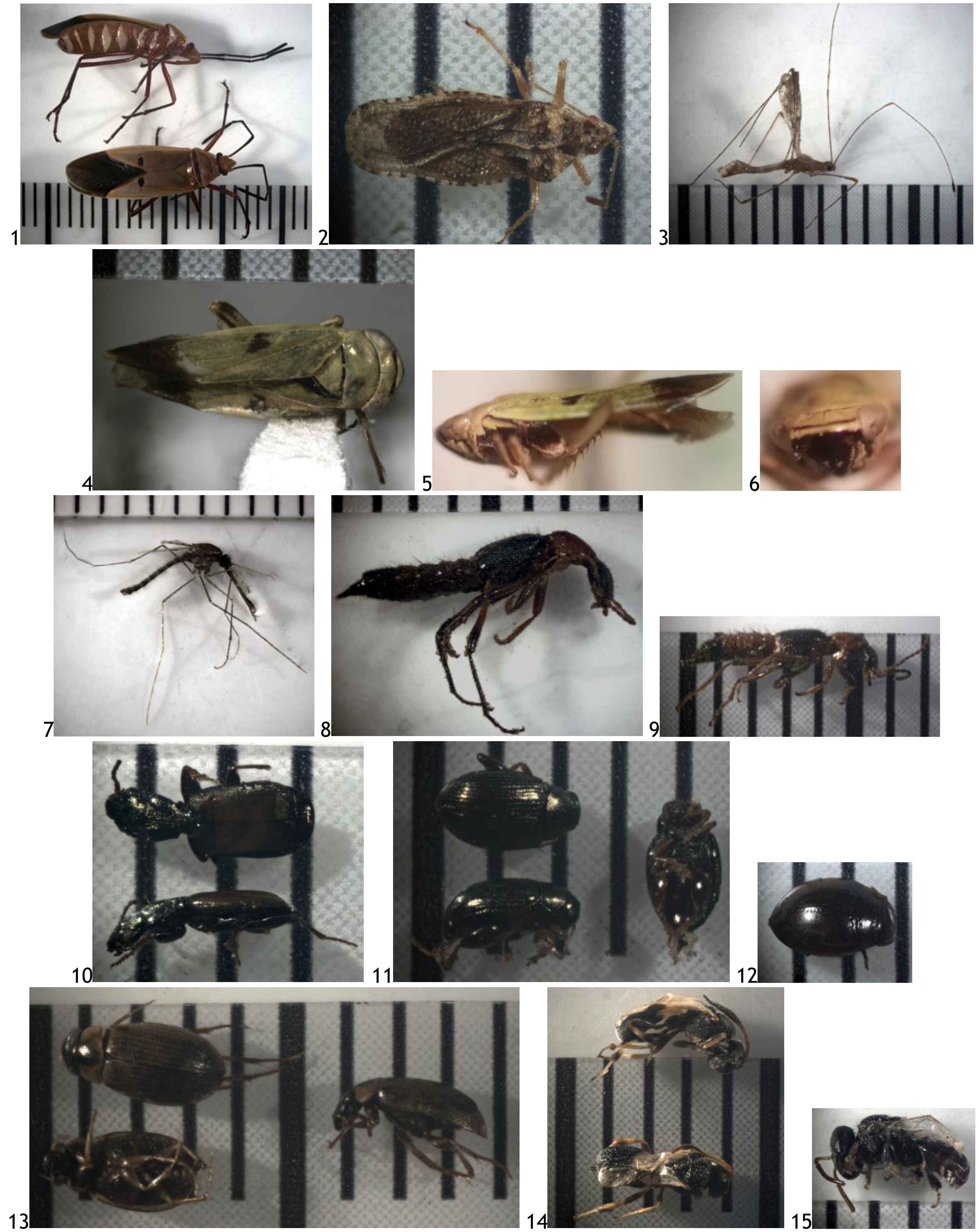

Plates 1-15. Selected species pictured with ruler; lines are 1mm. apart 1: *Dysdercus* sp., 2: *Cy. endeca*, 3: *M. nitidus*, 4-6: *N. modulatus*, 7: *A. coluzzii* (male), 8: *P. sabeus*, 9: *P. fuscipes*, 10: *Z. rhytidera*, 11: *Ch. coletta*, 12: *Hydrovatus* sp., 13: *Berosus* sp., 14: *Microchelonus* sp., 15: *Hypotrigona* sp.

Figure S2. L-shape distributions of the number of insects per panel (histogram) and kernel density function (line) for the selected taxa. Maximum value of the X-axis was truncated to 15 (some values were higher, see Max value, inset). N and Max denote the total and the maximum density/panel in 220 sticky nets.

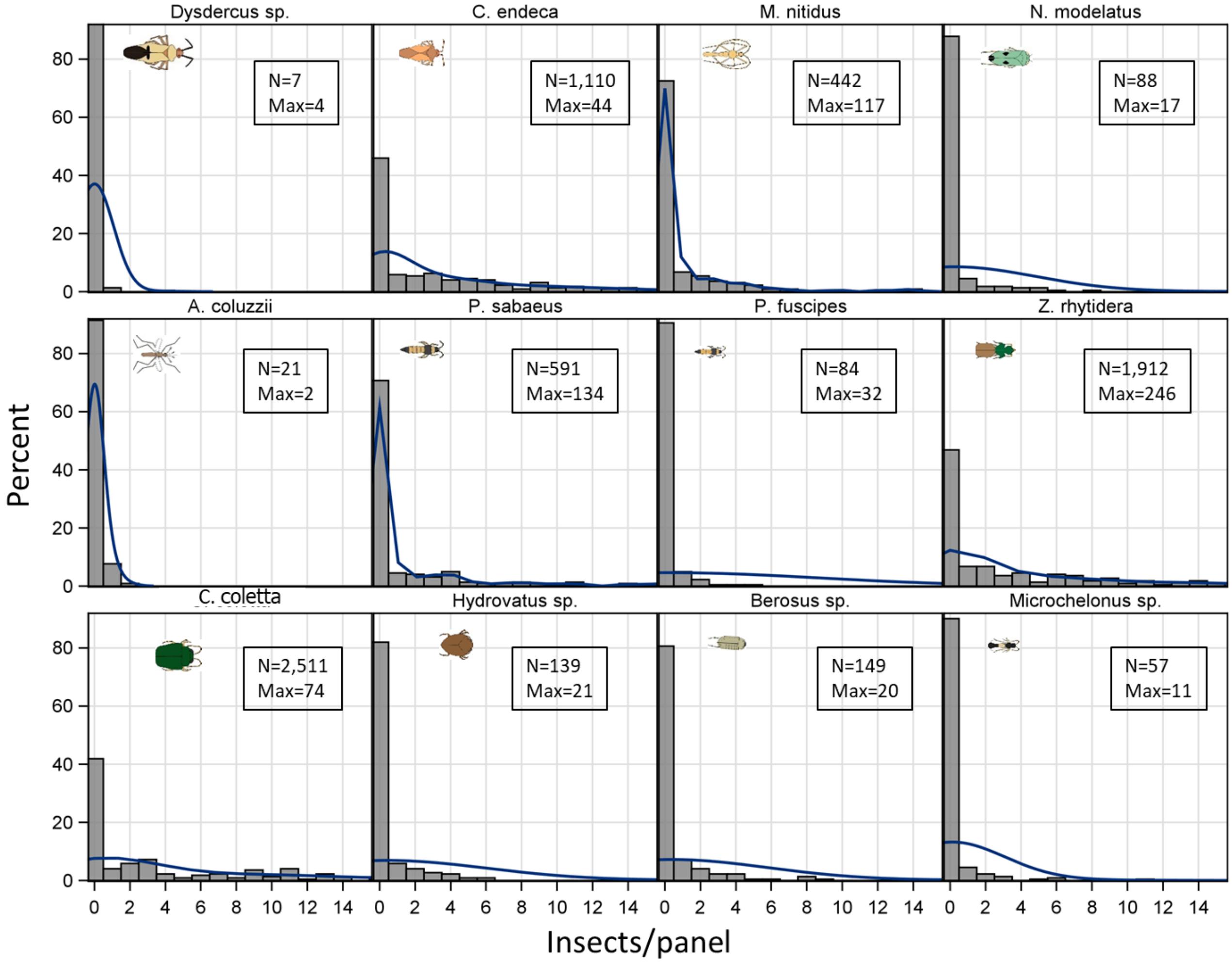

.

Figure S3. Seasonal variation in total-insect density/panel (note: sampling in 2013 ended in August). Inset: Relationship between panel density and aerial density (log scales). Pearson correlation coefficient (N reflects values larger than 0).

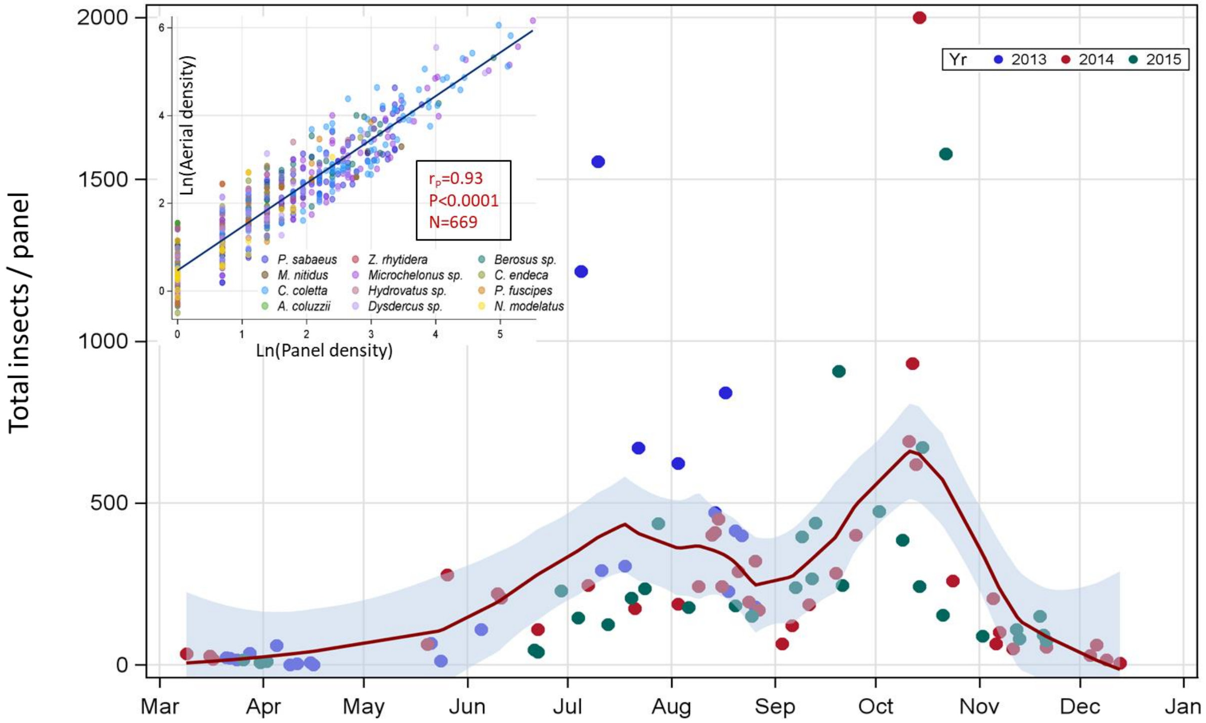

Figure S4. Distribution of mean nightly wind speed at flight height. Estimates are based on MERRA2 database and matched to the nearest panel altitude (50m, 70m and 200 m agl, see text from details).

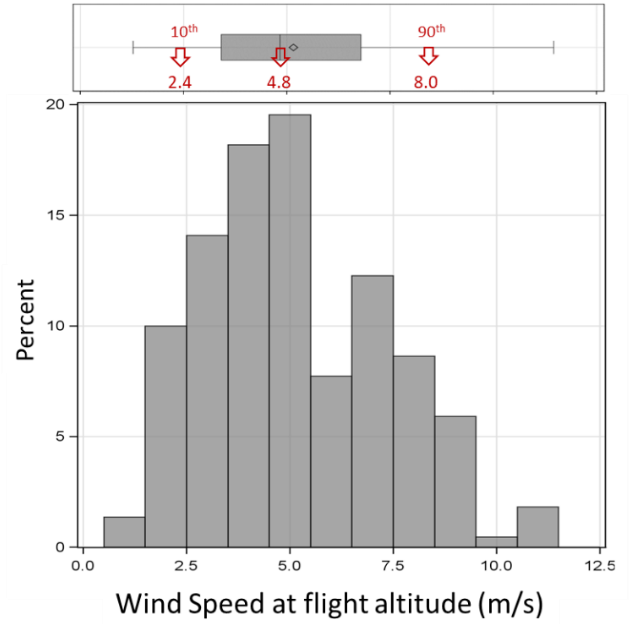

Figure S5: Weather conditions during high-altitude flight on the ground (red) and at flight height (blue) of each taxon. Weighted means (weighted by aerial density) and 95% CI on the ground and at flight height of (a) nightly temperature, (b) RH, and (c) wind speed are shown with corresponding wet season means (dashed lines labeled ‘W2m’ and ‘Air’) and year-round means at 2 m agl (dotted red lines labeled ‘Y2m’). Schematic insect silhouettes are not to scale.

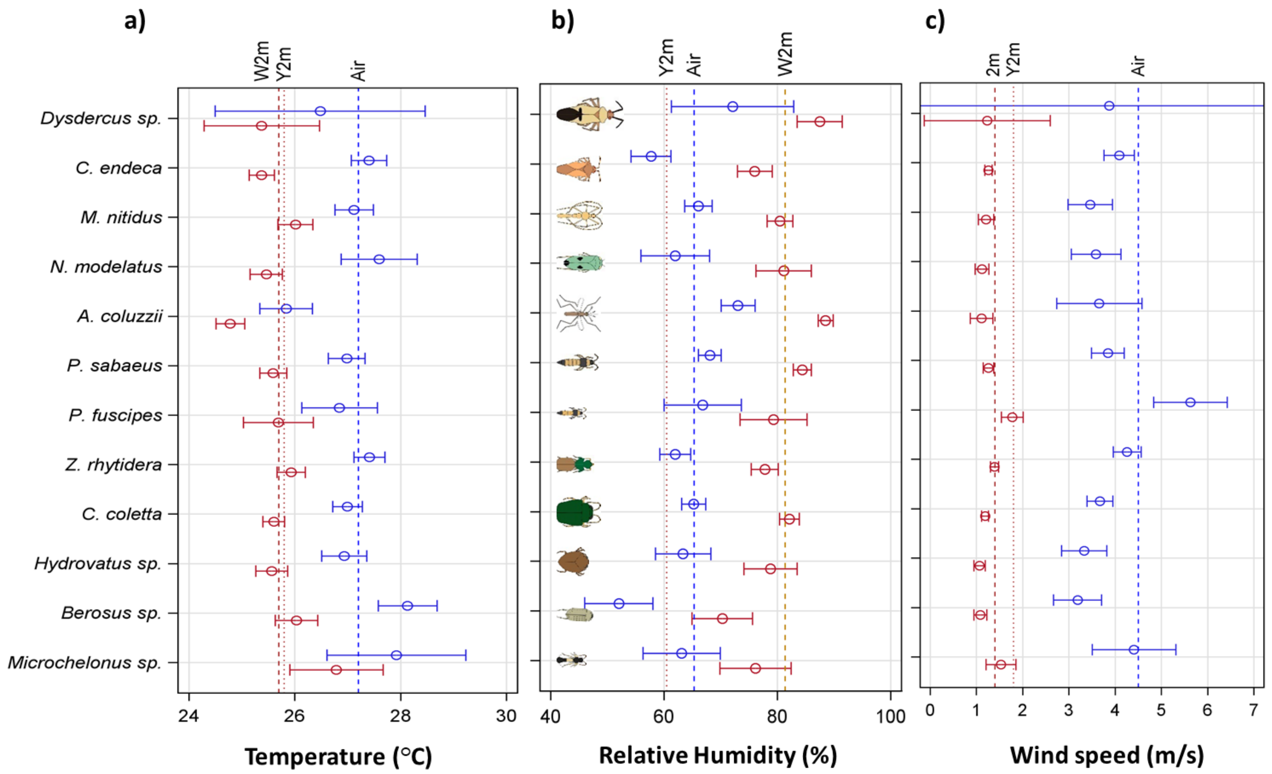

Table S1. List of identified insects from the project to-date.

Collection Site (	Collection Date	Height (m)	TubeIDOrig	Order	suborder	superfamily	family	subfamily	tribe	*genus*	*species*	TaxonomistNam
Markabougou, Mali	3-Aug-13	40	MB231A	Coleoptera	Adephaga	Caraboidea	Carabidae	Cicindelinae	Cicindelini	*Lophyra*	*senegalensis (Dejean, 19*	Schüle
Thierola, Mali (13.6	18-Aug-13	40	TB386	Coleoptera	Adephaga	Caraboidea	Carabidae	Harpalinae	Amblystomina	*Amblystomus*	*dispar (Basilewski, 1951)*	Schüle
Thierola, Mali (13.6	21-Mar-13	40	TB204	Coleoptera	Adephaga	Caraboidea	Carabidae	Harpalinae	Amblystomina	*Amblystomus*	*katanganus (Burgeon, 19*	Schüle
Thierola, Mali (13.6	7-Sep-15	120	TB960B	Coleoptera	Adephaga	Caraboidea	Carabidae	Harpalinae	Amblystomina	*Amblystomus*	*latefasciatus (Basilewsky*	Schüle
Suigima, Mali (14.1	21-May-13	40	SB111A	Coleoptera	Adephaga	Caraboidea	Carabidae	Harpalinae	Amblystomina	*Amblystomus*	*viridulus (Erichson, 1843*	Schüle
Suigima, Mali (14.1	21-May-13	40	SB111A	Coleoptera	Adephaga	Caraboidea	Carabidae	Harpalinae	Chlaeniini	*Callistochrous*	*baxi (Gory, 1833)*	Schüle
Suigima, Mali (14.1	21-May-13	40	SB111A	Coleoptera	Adephaga	Caraboidea	Carabidae	Harpalinae	Harpalinae	*Afromizonus*	*tecospilus (Basilewsky, 1*	Schüle
Suigima, Mali (14.1	21-May-13	40	SB111A	Coleoptera	Adephaga	Caraboidea	Carabidae	Harpalinae	Harpalini	*Egadroma*	*discriminatis (Basilewsky*	Schüle
Suigima, Mali (14.1	21-May-13	40	SB111A	Coleoptera	Adephaga	Caraboidea	Carabidae	Harpalinae	Harpalini	*Platymetopus*	*tesselatus (Basilewsky, 1*	Schüle
Thierola, Mali (13.6	7-Sep-15	120	TB960A	Coleoptera	Adephaga	Caraboidea	Carabidae	Lebiinae	Lebiini	*Metadromius*	*royi (Mateu, 1969)*	Schüle
Thierola, Mali (13.6	21-Mar-13	40	TB204	Coleoptera	Adephaga	Caraboidea	Carabidae	Lebiinae	Lebiini	*Singilis*	*bedimo (Anichtchenko, 2*	Schüle
Suigima, Mali (14.1	21-May-13	40	SB111A	Coleoptera	Adephaga	Caraboidea	Carabidae	Lebiinae	Lebiini	*Syntomus*	*submaculatus (Wollaston*	Schüle
Thierola, Mali (13.6	14-Aug-14	160	TB535B	Coleoptera	Adephaga	Caraboidea	Carabidae	Lebiinae	Odacanthini	*Lasiocera*	*nitidula (Dejean, 1831)*	Schüle
Thierola, Mali (13.6	7-Sep-15	120	TB960A	Coleoptera	Adephaga	Caraboidea	Carabidae	Lebiinae	Pentagonicini	*Pentagonica*	*elegans (Peringuey, 189*	Schüle
Thierola, Mali (13.6	7-Sep-15	120	TB960A	Coleoptera	Adephaga	Caraboidea	Carabidae	Trechinae	Bembidiini	*Polyderis*	*sp.*	Schüle
Suigima, Mali (14.1	21-Nov-14	160	SB574A	Coleoptera	Adephaga	Caraboidea	Carabidae			*Sphaerotachys*	*tetraspilus (Solsky, 1874*	Schüle
Markabougou, Mali (13.9128, -6.3425)			MB231A	Coleoptera	Adephaga	Dytiscoidea	Dytiscidae	Hydroporinae		*Hydrovatus*		Saverio Rocchi
Markabougou, Mali	20-Aug-13	40	MB255A-CO-2	Coleoptera	Adephaga	Dytiscoidea	Dytiscidae	Laccophilinae	Laccophilini	*Laccophilus*		Warren Steiner
Markabougou, Mali	13-Aug-14	190	MB395A-CO-2	Coleoptera	Adephaga	Dytiscoidea	Dytiscidae					Warren Steiner
Thierola, Mali (13.6	28-Oct-14	160	TB682A-CO-1	Coleoptera	Adephaga	Caraboidea	Carabidae	Brachininae	Brachinini	*Brachinus*	*prob. dorsalis Dejean*	Lourdes Chamorr
Thierola, Mali (13.6	4-Jul-15		TB820A	Coleoptera	Adephaga	Caraboidea	Carabidae	Lebiinae	Lebiini	*Zolotarevskyella*	*prob. rhytidodera (Csiki)*	Lourdes Chamorr
Thierola, Mali (13.6	7-Sep-15	120	TB960A	Coleoptera	Adephaga	Caraboidea	Carabidae	Trechinae	Bembidiini	*Tachyura*	*biplagiatus (Dejean, 183*	Schüle
Thierola, Mali (13.6	7-Sep-15	120	TB960A	Coleoptera	Adephaga	Caraboidea	Carabidae	Trechinae	Bembidiini	*Tachyura*	*cfr. vagans (Peringuey, 1*	Schüle
Thierola, Mali (13.6	7-Sep-15	120	TB960A	Coleoptera	Adephaga	Caraboidea	Carabidae	Trechinae	Bembidiini	*Tachyura*	*fumicata (Motschulsky, 1*	Schüle
Thierola, Mali (13.6	18-Aug-13	40	TB386	Coleoptera	Adephaga	Caraboidea	Carabidae	Trechinae	Bembidiini	*Tachyura*	*spec.*	Schüle
Suigima, Mali (14.1	7-Jul-14	40	SB286A-CO-8	Coleoptera	Adephaga	Caraboidea	Carabidae					Leonid Friedman
Thierola, Mali (13.6583, -7.2155)			TB669A	Coleoptera	Polyphaga	Bostrichoidea	Bostrichidae					Laura Verú
Suigima, Mali (14.1	7-Jul-14	40	SB286A-CO-8	Coleoptera	Polyphaga	Chrysomeloidea	Brentidae	Galerucinae				Leonid Friedman
Thierola, Mali (13.6583, -7.2155)			TB21A-CO-1	Coleoptera	Polyphaga	Chrysomeloidea	Chrysomelidae	Bruchinae	Pachymerini	*Caryedon?*		Laura Verú
Thierola, Mali (13.6	13-Aug-14	190	TB533B-CO-5	Coleoptera	Polyphaga	Chrysomeloidea	Chrysomelidae	Bruchinae				Leonid Friedman
Markabougou, Mali	15-Oct-14		MB446A-CO-1	Coleoptera	Polyphaga	Chrysomeloidea	Chrysomelidae	Criocerinae				Lourdes Chamorr
Markabougou, Mali	20-Aug-13	40	MB255A-CO-2	Coleoptera	Polyphaga	Chrysomeloidea	Chrysomelidae	Eumolpinae				Lourdes Chamorr
Markabougou, Mali	7-Jul-14	120	MB320A	Coleoptera	Polyphaga	Chrysomeloidea	Chrysomelidae	Galerucinae	Alticini	*Aphthona*	*laevissima (Wollaston)*	Maurizio Biondi
Markabougou, Mali	7-Jul-14	120	MB320A	Coleoptera	Polyphaga	Chrysomeloidea	Chrysomelidae	Galerucinae	Alticini	*Aphthona*	*signatifrons (Wollaston)*	Maurizio Biondi
Markabougou, Mali	13-Aug-14	190	MB395B-CO-3	Coleoptera	Polyphaga	Chrysomeloidea	Chrysomelidae	Galerucinae	Alticini	*Aphthona*	*sp. 1*	Furth
Markabougou, Mali	7-Jul-14	120	MB320A	Coleoptera	Polyphaga	Chrysomeloidea	Chrysomelidae	Galerucinae	Alticini	*Aphthona*	*whitfieldi (Bryant)*	Maurizio Biondi
Markabougou, Mali	14-Aug-14	190	MB398B	Coleoptera	Polyphaga	Chrysomeloidea	Chrysomelidae	Galerucinae	Alticini	*Chaetocnema*	*coletta (Bechyné)*	Maurizio Biondi
Markabougou, Mali	24-Oct-14	120	MB474A	Coleoptera	Polyphaga	Chrysomeloidea	Chrysomelidae	Galerucinae	Alticini	*Longitarsus*	*sp. 1*	Maurizio Biondi
Markabougou, Mali	7-Jul-14	120	MB320A	Coleoptera	Polyphaga	Chrysomeloidea	Chrysomelidae	Galerucinae	Alticini	*Longitarsus*	*sp. 2*	Maurizio Biondi
Suigima, Mali (14.1	10-Aug-14	190	SB362A-CO-9	Coleoptera	Polyphaga	Chrysomeloidea	Chrysomelidae	Galerucinae	Alticini			Maurizio Biondi
Markabougou, Mali	9-Aug-14	190	MB386A	Coleoptera	Polyphaga	Chrysomeloidea	Chrysomelidae	Galerucinae	Luperini	*Afromaculepta*	*decemmaculata*	Thomas Wagner
Thierola, Mali (13.6	16-Mar-14	40	TB416A-CO-4	Coleoptera	Polyphaga	Chrysomeloidea	Chrysomelidae	Galerucinae	Luperini	*Monolepta*	*sp. 1*	Furth
Thierola, Mali (13.6583, -7.2155)			TB392A-CO-3	Coleoptera	Polyphaga	Chrysomeloidea	Chrysomelidae	Galerucinae	Luperini	*Monolepta*	*sp. 2*	Furth
Thierola, Mali (13.6	14-Oct-14	120	TB639A	Coleoptera	Polyphaga	Chrysomeloidea	Chrysomelidae	Galerucinae	Luperini	*Monolepta*	*sp. 3*	Furth
Thierola, Mali (13.6	5-Jul-13	40	TB321A	Coleoptera	Polyphaga	Chrysomeloidea	Chrysomelidae	Galerucinae		*Panafrolepta*	*dahlmani*	Thomas Wagner
Suigima, Mali (14.1	17-Aug-13	160	SB179A	Coleoptera	Polyphaga	Cucujoidea	Coccinellidae	Coccidulini				Shockley
Markabougou, Mali	15-Oct-14		MB446A-CO-2	Coleoptera	Polyphaga	Cucujoidea	Coccinellidae					
Markabougou, Mali	13-Aug-14	190	MB395A-CO-2	Coleoptera	Polyphaga	Cucujoidea	Nitidulidae					Warren Steiner
Suigima, Mali (14.1	11-Sep-14	120	SB444A-CO-1	Coleoptera	Polyphaga	Cucujoidea	Phalacridae					Warren Steiner
Thierola, Mali (13.6	20-May-14		TB426B	Coleoptera	Polyphaga	Curculionoidea	Attelabidae	Rhynchitinae			*sp. 1*	Lourdes Chamorr
Markabougou, Mali	15-Aug-14	160	MB397A-CO-9	Coleoptera	Polyphaga	Curculionoidea	Brentidae	Nanophyinae		*Nanophyes*	*poss. errans*	Lourdes Chamorr
Thierola, Mali (13.6	10-Aug-14	190	TB527A-CO-5	Coleoptera	Polyphaga	Curculionoidea	Brentidae	Nanophyinae		*Nanophyes*	*sp. 1*	Lourdes Chamorr
Thierola, Mali (13.6	18-Aug-13	40	TB386A	Coleoptera	Polyphaga	Curculionoidea	Brentidae	Nanophyinae		*Nanophyes*	*sp. 2*	Lourdes Chamorr
Suigima, Mali (14.1	11-Sep-14	120	SB444A-CO-6	Coleoptera	Polyphaga	Curculionoidea	Brentidae	Apioninae				Lourdes Chamorr
Suigima, Mali (14.1	7-Sep-15		SB725	Coleoptera	Polyphaga	Curculionoidea	Curculionidae	Bagoinae		*Bagous*	*sp. 1*	Lourdes Chamorr
Thierola, Mali (13.6	16-Mar-14		TB418A	Coleoptera	Polyphaga	Curculionoidea	Curculionidae	Baridinae			*sp. 1*	Lourdes Chamorr
Thierola, Mali (13.6	17-Mar-14	120	TB420A	Coleoptera	Polyphaga	Curculionoidea	Curculionidae	Baridinae	Baridini	*Baris*	*picturatus*	Lourdes Chamorr
Suigima, Mali (14.1	21-Nov-16	160	SB574A-CO-11	Coleoptera	Polyphaga	Curculionoidea	Curculionidae	Baridinae			*sp. 3*	Lourdes Chamorr
Markabougou, Mali	22-Aug-13		MB262A	Coleoptera	Polyphaga	Curculionoidea	Curculionidae	Brachycerinae			*sp. 1*	Lourdes Chamorr
Suigima, Mali (14.1	7-Jul-14		SB288A	Coleoptera	Polyphaga	Curculionoidea	Curculionidae	Brachycerinae			*sp. 2*	Lourdes Chamorr
Thierola, Mali (13.6	7-Sep-15		TB960A	Coleoptera	Polyphaga	Curculionoidea	Curculionidae	Brachycerinae			*sp. 3*	Lourdes Chamorr
Suigima, Mali (14.1	20-Jul-15	190	SB662A	Coleoptera	Polyphaga	Curculionoidea	Curculionidae	Ceutorhynchinae	Ceutorhynchini	*Nr. Neocoeliodes*	*sp. 1*	Lourdes Chamorr

Thierola, Mali (13.6	13-Nov-15	120	TB1095A-CO-17	Coleoptera	Polyphaga	Curculionoidea	Curculionidae	Conoderinae		*Lobotrachelus*	*sp. 1*	Lourdes Chamorr
Thierola, Mali (13.6	6-Sep-14	190	TB593A-CO-19	Coleoptera	Polyphaga	Curculionoidea	Curculionidae	Curculioninae	Ochyromerini	*Endaeus*	*sp. 1*	Lourdes Chamorr
Suigima, Mali (14.1	10-Jul-13	120	SB127A-CO-5	Coleoptera	Polyphaga	Curculionoidea	Curculionidae	Curculioninae	Rhamphini		*sp. 1*	Lourdes Chamorr
Thierola, Mali (13.6	5-Jul-13	40	TB321A	Coleoptera	Polyphaga	Curculionoidea	Curculionidae	Curculioninae	Smicronychini	*Afrosmicronyx*	*dorsomaculatus (Julien H*	Lourdes Chamorr
Markabougou, Mali	26-Mar-15	190	MB544A-CO-6	Coleoptera	Polyphaga	Curculionoidea	Curculionidae	Curculioninae	Smicronychini	*Afrosmicronyx*	*umbrinus*	Lourdes Chamorr
Thierola, Mali (13.6	9-Mar-14	160	TB397A	Coleoptera	Polyphaga	Curculionoidea	Curculionidae	Curculioninae	Smicronychini	*Sharpia*	*bella*	Lourdes Chamorr
Suigima, Mali (14.1	7-Jul-14	160	SB288A	Coleoptera	Polyphaga	Curculionoidea	Curculionidae	Curculioninae	Smicronychini	*Smicronyx*	*gossypii (Haran)*	Lourdes Chamorr
Markabougou, Mali	15-Aug-14	160	MB397A-CO-8	Coleoptera	Polyphaga	Curculionoidea	Curculionidae	Curculioninae	Smicronychini	*Smicronyx*	***zambianus n. sp. (Hara***	Julien Haran & Lo
Suigima, Mali (14.1	22-May-13	160	SB113A-CO-7	Coleoptera	Polyphaga	Curculionoidea	Curculionidae	Lixinae	Lixini	*Microlarinus*	*sp.*	Lourdes Chamorr
Suigima, Mali (14.1666, -7.2332)			SB573	Coleoptera	Polyphaga	Curculionoidea	Curculionidae	Scolytinae				Laura Verú
Markabougou, Mali	13-Aug-14	190	MB395A-CO-8	Coleoptera	Polyphaga	Curculionoidea	Curculionidae					Lourdes Chamorr
Markabougou, Mali	22-Aug-13		MB262A	Coleoptera	Polyphaga	Curculionoidea	Erorhinidae	Erirhininae			*sp. 1*	Lourdes Chamorr
Markabougou, Mali	7-Jul-14		MB319A	Coleoptera	Polyphaga	Curculionoidea	Brentidae	Apioninae			*#1 female*	Lourdes Chamorr
Thierola, Mali (13.6	5-Jul-13		TB322C	Coleoptera	Polyphaga	Curculionoidea	Brentidae	Apioninae			*#1 male*	Lourdes Chamorr
Markabougou, Mali	25-Sep-14		MB429E	Coleoptera	Polyphaga	Curculionoidea	Brentidae	Apioninae			*#2 female*	Lourdes Chamorr
Thierola, Mali (13.6	5-Jul-13		TB322A	Coleoptera	Polyphaga	Curculionoidea	Brentidae	Apioninae			*#2 male*	Lourdes Chamorr
Thierola, Mali (13.6	11-Oct-14		TB630A	Coleoptera	Polyphaga	Curculionoidea	Brentidae	Apioninae			*#3*	Lourdes Chamorr
Thierola, Mali (13.6	11-Oct-14		TB630A	Coleoptera	Polyphaga	Curculionoidea	Brentidae	Apioninae			*#4*	Lourdes Chamorr
Thierola, Mali (13.6	21-Jul-14		TB502B	Coleoptera	Polyphaga	Curculionoidea	Brentidae	Apioninae			*#5*	Lourdes Chamorr
Markabougou, Mali	24-Oct-14		MB474A	Coleoptera	Polyphaga	Curculionoidea	Brentidae	Apioninae			*#6*	Lourdes Chamorr
Thierola, Mali (13.6	7-Jul-14		TB463D	Coleoptera	Polyphaga	Curculionoidea	Brentidae	Apioninae			*#7*	Lourdes Chamorr
Markabougou, Mali	7-Jul-14		MB319A	Coleoptera	Polyphaga	Curculionoidea	Brentidae	Apioninae			*#8*	Lourdes Chamorr
Thierola, Mali (13.6	22-Oct-15		TB1056B	Coleoptera	Polyphaga	Curculionoidea	Brentidae	Apioninae		*Conapium #1*		Lourdes Chamorr
Thierola, Mali (13.6	22-Oct-15		TB1056B	Coleoptera	Polyphaga	Curculionoidea	Brentidae	Apioninae		*Conapium #2*		Lourdes Chamorr
Markabougou, Mali	7-Jul-14		MB319A	Coleoptera	Polyphaga	Curculionoidea	Curculionidae	Curculioninae			*sp. 1*	Lourdes Chamorr
Thierola, Mali (13.6	7-Jul-14		TB463D	Coleoptera	Polyphaga	Curculionoidea	Brentidae	Apioninae	Piezotrachelini		*#1*	Lourdes Chamorr
Markabougou, Mali	26-Aug-13		MB272A	Coleoptera	Polyphaga	Curculionoidea	Brentidae	Apioninae	Pizeotrachelini		*#2*	Lourdes Chamorr
Markabougou, Mali	15-Aug-14	160	MB397A-CO-1	Coleoptera	Polyphaga	Elateroidea	Elateridae					Warren Steiner/Lo
Suigima, Mali (14.1	11-Sep-14	120	SB444A-CO-1	Coleoptera	Polyphaga	Hydrophiloidea	Hydrophilidae	Hydrophilinae	Berosini	*Berosus*		Warren Steiner
Markabougou, Mali	15-Aug-14	160	MB397A-CO-1	Coleoptera	Polyphaga	Hydrophiloidea	Hydrophilidae					Warren Steiner
Thierola, Mali (13.6	3-Aug-14		TB512A-CO-2	Coleoptera	Polyphaga	Scarabaeoidea	Scarabaeidae					Lourdes Chamorr
Markabougou, Mali	13-Aug-14	190	MB395A-CO-2	Coleoptera	Polyphaga	Staphylinoidea	Staphilinidae	Pselaphinae				Warren Steiner
Suigima, Mali (14.1	10-Aug-14	190	SB362A-CO-9	Coleoptera	Polyphaga	Staphylinoidea	Staphylindae					Leonid Friedman
Suigima, Mali (14.1	19-Jul-14	90	SB326A-CO-1	Coleoptera	Polyphaga	Staphylinoidea	Staphylinidae	Aleocharinae	Lomechusini	*Diplopleurus*		Dr. Jan Klimaszev
Thierola, Mali (13.6	13-Aug-14	160	TB532A-CO-1	Coleoptera	Polyphaga	Staphylinoidea	Staphylinidae	Aleocharinae	Lomechusini	*Paramyrmoecia*	bipustulata	Dr. Jan Klimaszev
Thierola, Mali (13.6	05-Aug-13	160	TB374A-CO-3	Coleoptera	Polyphaga	Staphylinoidea	Staphylinidae	Aleocharinae	Lomechusini	*Zyras (Ctenodonia)*		Dr. Jan Klimaszev
Thierola, Mali (13.6	13-Aug-14	160	TB532A-CO-1	Coleoptera	Polyphaga	Staphylinoidea	Staphylinidae	Paederinae	Paederini	*Paederus*	*fuscipes (Curtis)*	Dr. Frank
Suigima, Mali (14.1	06-Sep-14	190	SB431A-CO-1	Coleoptera	Polyphaga	Staphylinoidea	Staphylinidae	Paederinae	Paederini	*Paederus*	*sabaeus (Erichson)*	Dr. Frank
Thierola, Mali (13.6	13-Aug-14	160	TB532A-CO-1	Coleoptera	Polyphaga	Staphylinoidea	Staphylinidae	Paederinae	Paederini	*Paederus*		Dr.Frank
Thierola, Mali (13.6	13-Aug-14	160	TB532A-CO-1	Coleoptera	Polyphaga	Staphylinoidea	Staphylinidae	Staphylininae	Staphylinini	*Gabronthus*	*maritimus (Motschulsky)*	Dr. Frank
Thierola, Mali (13.6	14-Sep-14	160	TB616A-CO-1	Coleoptera	Polyphaga	Staphylinoidea	Staphylinidae	Staphylininae	Staphylinini	*Gabronthus*		Dr. Frank
Markabougou, Mali	10-Jul-13	120	MB214A-CO-3	Coleoptera	Polyphaga	Staphylinoidea	Staphylinidae	Staphylininae	Staphylinini	*Philonthus*		Dr. Frank
Thierola, Mali (13.6	10-Aug-14	190	TB527A-CO-6	Coleoptera	Polyphaga	Tenebrionidae	Aderidae					Warren Steiner
Suigima, Mali (14.1	11-Sep-14	120	SB444A-CO-7	Coleoptera	Polyphaga	Tenebrionidae	Anthicidae					Warren Steiner
Markabougou, Mali (13.9128, -6.3425)			MB476B	Coleoptera	Polyphaga	Tenebrionoidea	Mordellidae					Laura Verú
Markabougou, Mali	10-Jul-13	120	MB214A-DI-2	Diptera	Brachycera	Carnoidea	Chloropidae			*Epimadiza*		Amnon Freidberg
Suigima, Mali (14.1	10-Aug-14	190	SB362A-DI-1	Diptera	Brachycera	Carnoidea	Chloropidae					John Ismay
Markabougou, Mali	14-Aug-13	160	MB242A-DI-3	Diptera	Brachycera	Carnoidea	Milichiidae	Madizinae		*Phyllomyza*		Amnon Freidberg
Thierola, Mali (13.6583, -7.2155)			TB513B	Diptera	Brachycera	Diopsoidea	Diopsidae	Diopsinae	Diopsini	*Diopsis*		
Markabougou, Mali	14-Aug-14	190	MB398A-DI-3	Diptera	Brachycera	Empidoidea	Dolichopodidae					Igor Grichanov
Markabougou, Mali	13-Aug-14	160	MB394B-DI-3	Diptera	Brachycera	Ephydroidea	Curtonotidae			*Curtonotum*	*saheliense Tsacas, 1977*	Ashley Kirk-Sprigg
Thierola, Mali (13.6	6-Sep-14	160	TB593A-DI-5	Diptera	Brachycera	Ephydroidea	Drosophilidae	Steganinae	Steganini	*Leucophenga*		Amnon Freidberg
Thierola, Mali (13.6	13-Aug-14	190	TB533A-DI-4	Diptera	Brachycera	Ephydroidea	Drosophilidae					Shane McEvey
Markabougou, Mali	7-Nov-14	160	MB493A-DI-2	Diptera	Brachycera	Ephydroidea	Ephydridae	Discomyzinae	Psilopini	*Psitola*		Amnon Freidberg
Thierola, Mali (13.6	6-Sep-14	160	TB593A-DI-4	Diptera	Brachycera	Ephydroidea	Ephydridae	Hydrellinae		*Notiphila*		Amnon Freidberg
Markabougou, Mali	10-Jul-13	120	MB214A-DI-1	Diptera	Brachycera	Ephydroidea	Ephydridae					Tadeusz Zatwarni
Markabougou, Mali	19-Jul-14	190	MB353A-DI-1	Diptera	Brachycera	Lauxanioidea	Lauxaniidae					Stephen Gaimari
Thierola, Mali (13.6	13-Aug-14	190	TB533B-DI-4	Diptera	Brachycera	Muscoidea	Anthomyiidae					Verner Michelsen
Markabougou, Mali	14-Aug-13	40	MB240A-DI-4	Diptera	Brachycera	Muscoidea	Muscidae	Muscinae	Muscini	*Musca*		Amnon Freidberg
Markabougou, Mali	14-Aug-13	40	MB240A-DI-4	Diptera	Brachycera	Muscoidea	Muscidae	Muscinae	Stomoxyini			Amnon Freidberg
Markabougou, Mali	14-Aug-14	190	MB398A-DI-2	Diptera	Brachycera	Muscoidea	Muscidae	Phaoniinae	Atherigonini	*Atherigona*		Burgert Muller
Markabougou, Mali	14-Aug-13	40	MB240A-DI-4	Diptera	Brachycera	Muscoidea	Muscidae					Marcia Couri
Markabougou, Mali	22-Aug-13	40	MB261A-DI-1	Diptera	Brachycera	Oestroidea	Calliphoridae	Chrysomyiinae	Rhiniini	*Rhynchomyia*		Amnon Freidberg

Markabougou, Mali	14-Aug-13	40	MB240A-DI-2	Diptera	Brachycera	Oestroidea	Calliphoridae	Chrysomyiinae	Rhiniini			Amnon Freidberg
Markabougou, Mali	14-Aug-13	160	MB242A-DI-3	Diptera	Brachycera	Oestroidea	Rhiniidae					Knut Rognes
Markabougou, Mali	14-Aug-13	40	MB240A-DI-4	Diptera	Brachycera	Oestroidea	Tachinidae					Pierfilippo Cerretti
Suigima, Mali (14.1	19-Jul-14	90	SB326A-DI-1	Diptera	Brachycera	Platypezoidea	Phoridae					Amnon Freidberg
Markabougou, Mali	10-Jul-13	120	MB214A-DI-1	Diptera	Brachycera	Sciomyzoidea	Sepsidae					Andrey Ozerov
Thierola, Mali (13.6	13-Aug-14	160	TB532A-DI-1	Diptera	Brachycera	Syrphoidea	Pipunculidae					Marc De Meyer
Thierola, Mali (13.6	13-Aug-14	190	TB533B-DI-3	Diptera	Brachycera	Tephritoidea	Lonchaeidae	Lonchaeinae		*Silba*		Amnon Freidberg
Thierola, Mali (13.6	24-Aug-14	190	TB563B-DI-4	Diptera	Brachycera	Tephritoidea	Platystomatidae					Andrew Whittingto
Thierola, Mali (13.6	6-Sep-14	160	TB593A-DI-4	Diptera	Brachycera	Tephritoidea	Tephritidae	Dacinae	Ceratidini	*Ceratitis*		Marc De Meyer
Markabougou, Mali	14-Aug-13	160	MB242A-DI-4	Diptera	Brachycera	Tephritoidea	Ulidiidae	Ulidiinae	Ulidiini	*Physiphora*		Elena Kameneva
Thierola, Mali (13.6	13-Aug-14	160	TB532B-DI-1	Diptera	Nematocera	Chironomoidea	Ceratopogonidae					Amnon Freidberg
Markabougou, Mali	14-Aug-13	160	MB242A-DI-2	Diptera	Nematocera	Chironomoidea	Chironomidae					Torbjørn Ekrem
Thierola, Mali (13.6	7-Jul-14	120	TB461A	Diptera	Nematocera	Culicomorpha	Simuliidae	Simuliinae		*Simulium*	*griseicolle*	Peter Adler
Thierola, Mali (13.6	6-Sep-14	190	TB593A	Hemiptera	Auchenorrhyncha	Fulgoroidea	Delphacidae	Delphacinae	Delphacini	*Leptodelphax*	*maculigera*	Charles Bartlett
Thierola, Mali (13.6	17-Oct-14	190	TB650A	Hemiptera	Auchenorrhyncha	Fulgoroidea	Delphacidae	Delphacinae	Delphacini	*Perkinsiella*	*dorsata*	Charles Bartlett
Thierola, Mali (13.6	9/14/2014	160	TB616A	Hemiptera	Auchenorrhyncha	Fulgoroidea	Delphacidae	Delphacinae	Delphacini	*Sogatella*	*albofimbriata*	Charles Bartlett
Markabougou, Mali (13.9128, -6.3425)			MB395B-HO-5	Hemiptera	Auchenorrhyncha	Fulgoroidea	Delphacidae	Delphacinae	Delphacini	*Sogatella*	*cf albofimbriata*	Charles Bartlett
Thierola, Mali (13.6	20-Aug-13	120	TB393A-HO-2	Hemiptera	Auchenorrhyncha	Fulgoroidea	Delphacidae	Delphacinae	Delphacini	*Sogatella*	*cf furcifera*	Charles Bartlett
Thierola, Mali (13.6	14-Sep-14	160	TB616A	Hemiptera	Auchenorrhyncha	Fulgoroidea	Delphacidae	Delphacinae	Delphacini	*Sogatella*	*nigeriensis*	Charles Bartlett
Thierola, Mali (13.6583, -7.2155)			TB462C	Hemiptera	Auchenorrhyncha	Fulgoroidea	Delphacidae	Delphacinae	Delphacini	*Sogatella*	*vibex*	Charles Bartlett
Thierola, Mali (13.6583, -7.2155)			TB616A-HO-3	Hemiptera	Auchenorrhyncha	Fulgoroidea	Delphacidae	Delphacinae	Delphacini	*Sogatella*		Charles Bartlett
Thierola, Mali (13.6	13-Aug-14	190	TB533A	Hemiptera	Auchenorrhyncha	Fulgoroidea	Delphacidae	Delphacinae	Delphacini	*Thriambus*	*strennus*	Charles Bartlett
Thierola, Mali (13.6	13-Aug-14	190	TB533A	Hemiptera	Auchenorrhyncha	Fulgoroidea	Delphacidae	Delphacinae	Delphacini	*Toya*	*ceresensis*	Charles Bartlett
Thierola, Mali (13.6	14-Sep-14	160	TB616A	Hemiptera	Auchenorrhyncha	Fulgoroidea	Delphacidae	Delphacinae	Delphacini	*Toya*	*tuberculosa*	Charles Bartlett
Thierola, Mali (13.6583, -7.2155)			TB527A-HO-1	Hemiptera	Auchenorrhyncha	Fulgoroidea	Delphacidae	Delphacinae	Delphacini			Charles Bartlett
Thierola, Mali (13.6	6-Sep-14	160	TB593A-HO-8	Hemiptera	Auchenorrhyncha	Fulgoroidea	Delphacidae	Stenocraninae		*Stenocranus*		Tatina Novoselsky
Thierola, Mali (13.6583, -7.2155)			TB426A	Hemiptera	Auchenorrhyncha	Fulgoroidea	Flatidae					Charles Bartlett
Thierola, Mali (13.6583, -7.2155)			TB650A	Hemiptera	Auchenorrhyncha	Fulgoroidea	Ricaniidae					Charles Bartlett
Thierola, Mali (13.6	13-Aug-14	160	TB532B	Hemiptera	Auchenorrhyncha	Membracoidea	Cicadellidae	Deltocephalinae	Chiasmini	*Exitianus*	*distanti*	Charles Bartlett
Thierola, Mali (13.6	3-Aug-14		TB512A-HO-1	Hemiptera	Auchenorrhyncha	Membracoidea	Cicadellidae	Deltocephalinae	Chiasmini	*Exitianus*		James Zahniser
Markabougou, Mali	7-Nov-14	160	MB493A	Hemiptera	Auchenorrhyncha	Membracoidea	Cicadellidae	Deltocephalinae	Chiasmini	*Nephotettix*	*modulatus*	Charles Bartlett
Thierola, Mali (13.6583, -7.2155)			TB752A-HO-4	Hemiptera	Auchenorrhyncha	Membracoidea	Cicadellidae	Deltocephalinae	Chiasmini	*Nephotettix*	*cf modulatus*	Charles Bartlett
Thierola, Mali (13.6	13-Aug-14	190	TB533A-HO-1	Hemiptera	Auchenorrhyncha	Membracoidea	Cicadellidae	Deltocephalinae	Paralimnini	*Psammotettix*		Tatina Novoselsky
Thierola, Mali (13.6	24-Aug-14	190	TB563A	Hemiptera	Auchenorrhyncha	Membracoidea	Cicadellidae	Deltocephalinae	Paralimnini			James Zahniser
Thierola, Mali (13.6583, -7.2155)			TB650A	Hemiptera	Auchenorrhyncha	Membracoidea	Cicadellidae	Deltocephalinae	Vartini?			Charles Bartlett
Thierola, Mali (13.6583, -7.2155)			TB688A	Hemiptera	Auchenorrhyncha	Membracoidea	Cicadellidae					Charles Bartlett
Thierola, Mali (13.6	17-Oct-14	190	TB650A	Hemiptera	Heteroptera	Cimicoidea	Nabidae					Carsten Morkel
Thierola, Mali (13.6	14-Oct-15	120	TB1035A	Hemiptera	Heteroptera	Coreoidea	Rhopalidae					Carsten Morkel
Thierola, Mali (13.6	13-Aug-14	190	TB533B-HE-1	Hemiptera	Heteroptera	Coreoidea	Stenocephalidae			*Dicranocephalus*		Thomas Henry
Thierola, Mali (13.6583, -7.2155)			TB533A-HO-4	Hemiptera	Heteroptera	Corixoidea	Corixidae					Charles Bartlett
Thierola, Mali (13.6583, -7.2155)			TB534A	Hemiptera	Heteroptera	Gerroidea	Gerridae	Trepobatinae				
Thierola, Mali (13.6	5-Nov-14	160	TB688A	Hemiptera	Heteroptera	Gerroidea	Gerridae					Carsten Morkel
Thierola, Mali (13.6	19-Sep-14	120	TB627A	Hemiptera	Heteroptera	Gerroidea	Veliidae	Microveliinae	Microveliini	*Microvelia*	*sp.*	Andreas Krüger
Thierola, Mali (13.6	13-Aug-14	160	TB532A-PH-1	Hemiptera	Heteroptera	Hydrometroidea	Hydrometridae	Hydromerinae		*Hydrometra*		Tatina Novoselsky
Thierola, Mali (13.6	10-Aug-14	190	TB527A-HE-2	Hemiptera	Heteroptera	Lygaeoidea	Berytidae	Metacanthinae	Metacanthini	*Metacanthus*	*nitidus*	Carsten Morkel
Thierola, Mali (13.6	24-Aug-14	190	TB563A-HE-4	Hemiptera	Heteroptera	Lygaeoidea	Berytidae	Metacanthinae		*Yemma*		Tatina Novoselsky
Thierola, Mali (13	19-Sep-14	190	TB629A	Hemiptera	Heteroptera	Lygaeoidea	Geocoridae	Geocorinae		*Geocoris*	*aff. erytrops*	Andreas Krüger
Markabougou, Mali	19-Jul-14	190	MB353A-HE-5	Hemiptera	Heteroptera	Lygaeoidea	Lygaeidae	Orsillinae	Nysiini	*Nysius*		Thomas Henry
Suigima, Mali (14.1	24-Oct-14	190	SB488A-HE-3	Hemiptera	Heteroptera	Lygaeoidea	Lygaeidae	Rhyparochromina	Drymini	*Stilbocoris*		Tatina Novoselsky
Suigima, Mali (14.1	24-Oct-14	120	SB486A-HE-2	Hemiptera	Heteroptera	Lygaeoidea	Lygaeidae					Thomas Henry
Thierola, Mali (13.6	24-Aug-14	190	TB563A-HE-4	Hemiptera	Heteroptera	Lygaeoidea	Oxycarenidae			*Camptotelus*		Tatina Novoselsky
Thierola, Mali (13.6	13-Aug-14	160	TB532A-HE-1	Hemiptera	Heteroptera	Lygaeoidea	Phyparochromida	Rhyparochromina	Rhyparochromini	*Beosus*		Tatina Novoselsky
Thierola, Mali (13.6	14-Oct-15	120	TB1035A	Hemiptera	Heteroptera	Lygaeoidea	Rhyparochromida	Rhyparochromina	Myodochini	*cf. Paromius*		Carsten Morkel
Thierola, Mali (13.6	14-Oct-15	120	TB1035A	Hemiptera	Heteroptera	Lygaeoidea	Rhyparochromida	Rhyparochromina	Myodochini	*Paromius*	*gracilis*	Carsten Morkel
Suigima, Mali (14.1	24-May-13	40	SB120A	Hemiptera	Heteroptera	Lygaeoidea	Rhyparochromida	Rhyparochromina	Ozophorini	*Ethaltomarus*	*glabrosus (Scudder)*	Andreas Krüger/E
Markabougou, M	21-Jul-14	190	MB359A	Hemiptera	Heteroptera	Lygaeoidea	Rhyparochromida	Rhyparochromina	Stygnocorini	*cf.Lasiosomus*	*sp.*	Andreas Krüger
Suigima, Mali (14.1	19-Jul-14	120	SB327A-HE-2	Hemiptera	Heteroptera	Lygaeoidea	Rhyparochromidae					Thomas Henry
Thierola, Mali (13	19-Sep-14	190	TB629A	Hemiptera	Heteroptera	Miroidea	Miridae					Andreas Krüger
Thierola, Mali (13	14-Aug-14	160	TB535B	Hemiptera	Heteroptera	Notonectoidea	Notonectidae	Anisopinae		*Anisops*	*spec.*	Andreas Krüger
Markabougou, Mali (13.9128, -6.3425)			MB261A-HO-4	Hemiptera	Heteroptera	Notonectoidea	Notonectidae					Charles Bartlett
Suigima, Mali (14.1	24-Oct-14	190	SB488A-HE-3	Hemiptera	Heteroptera	Pentatomoidea	Cydnidae	Cydnidae	Geotomini	*Dallasiellus*	*discrepans*	Tatina Novoselsky

Suigima, Mali (14.1	24-Oct-14	120	SB486A-HE-1	Hemiptera	Heteroptera	Pentatomoidea	Cydnidae	Cydninae	Geotomini	*Aethus*		Thomas Henry
Suigima, Mali (14.1	14-Oct-15	120	SB795A	Hemiptera	Heteroptera	Pentatomoidea	Cydnidae					Carsten Morkel
Suigima, Mali (14.1	24-Oct-14	120	SB486A-HE-3	Hemiptera	Heteroptera	Pentatomoidea	Pentatomidae	Pentatominae	Antestini	*Adria*	*parvula*	Thomas Henry
Suigima, Mali (14.1	14-Oct-15	120	SB795A	Hemiptera	Heteroptera	Pentatomoidea	Pentatomidae					Carsten Morkel
Thierola, Mali (13.6	14-Jul-14	190	TB484A	Hemiptera	Heteroptera	Pyrrhocoroidea	Pyrrhocoridae	Pyrrhocorinae		*Dysdercus*	*sp.*	Andreas Krüger
Suigima, Mali (14.1	7-Jul-14	40	SB286A-HE-5	Hemiptera	Heteroptera	Reduvioidea	Reduviidae					Carsten Morkel
Thierola, Mali (13.6	14-Sep-14	160	TB616A-HE-1	Hemiptera	Heteroptera	Tingoidea	Tingidae	Tingidae	Tingini	*Cysteochila*	*endeca*	Thomas Henry
Suigima, Mali (14.1	7-Jul-14	40	SB286A-HE-6	Hemiptera	Heteroptera	Tingoidea	Tingidae	Tinginae	Tingini	*Dictyla*		Tatina Novoselsky
Thierola, Mali (13.6	5-Nov-14	160	TB688A	Hemiptera	Heteroptera	Tingoidea	Tingidae					Carsten Morkel
Markabougou, Mali (13.9128, -6.3425)			MB416B	Hemiptera	Sternorrhyncha	Aphidoidea	Aphididae					Laura Verú
Thierola, Mali (13.6583, -7.2155)			TB533A-HO-4	Hemiptera	Sternorrhyncha	Psylloidea						Charles Bartlett
Markabougou, Mali	21-Aug-14	190	MB416B	Hymenoptera	Apocrita	Apoidea	Apidae	Apinae	Meliponini	*Hypotrigona*		Corey Smith/John
Thierola, Mali (13.6	3-Aug-14		TB512A-HY-2	Hymenoptera	Apocrita	Apoidea	Crabronidae					Elijah Talamas
Thierola, Mali (13.6	24-Aug-14	190	TB563B-HY-2	Hymenoptera	Apocrita	Apoidea	Megachilidae					Elijah Talamas
Thierola, Mali (13.6	20-Aug-13	40	TB392A-HY-2	Hymenoptera	Apocrita	Apoidea	Sphecidae					Elijah Talamas
Markabougou, Mali	15-Oct-14		MB446A-HY-1	Hymenoptera	Apocrita	Apoidea						Elijah Talamas
				Hymenoptera	Apocrita	Chalcidoidea	Chalcicidae	Epitraninae		*Epitranus*		Bob Copeland
Thierola, Mali (13.6583, -7.2155)			TB562B	Hymenoptera	Apocrita	Chalcidoidea	Eulophidae					Jason Mottern
Markabougou, Mali	20-Aug-13	40	MB255A-HY-1	Hymenoptera	Apocrita	Chalcidoidea	Eupelmidae					Elijah Talamas
Thierola, Mali (13.6583, -7.2155)			TB838A	Hymenoptera	Apocrita	Chalcidoidea	Eurytomidae					
Thierola, Mali (13.6	24-Aug-14	190	TB563A-HY-3	Hymenoptera	Apocrita	Chalcidoidea						Elijah Talamas
Thierola, Mali (13.6	24-Aug-14	190	TB563B-HY-3	Hymenoptera	Apocrita	Chrysidoidea	Bethylidae					Elijah Talamas
Suigima, Mali (14.1666, -7.2332)			SB053	Hymenoptera	Apocrita	Chrysidoidea	Chrysididae					Laura Verú
Thierola, Mali (13.6	22-Oct-15	120	TB1056B	Hymenoptera	Apocrita	Chrysidoidea	Dryinidae	Gonatopodinae		*Gonatopus*		Massimo Olmi
Thierola, Mali (13.6583, -7.2155)			TB608A	Hymenoptera	Apocrita	Cynipoidea	Figitidae	Eucoilinae		*Afrostilba*		Jason Mottern
Markabougou, Mali	14-Aug-14	160	MB397A-HY-1	Hymenoptera	Apocrita	Diaprioidea	Diapriidae	Diapriinae	Psilini	*Coptera*		Elijah Talamas
Markabougou, Mali	15-Aug-14	160	MB397A-HY-1	Hymenoptera	Apocrita	Diaprioidea	Diapriidae					Elijah Talamas
Markabougou, Mali	19-Jul-14	160	MB352A-HY-4	Hymenoptera	Apocrita	Ichneumonoidea	Braconidae	Cheloninae				Elijah Talamas
Markabougou, Mali	19-Jul-14	160	MB352A-HY-1	Hymenoptera	Apocrita	Ichneumonoidea	Braconidae					Elijah Talamas
Thierola, Mali (13.6583, -7.2155)			TB608A	Hymenoptera	Apocrita	Ichneumonoidea	Ichneumonidae	Tersilochinae				Robert Kula
Thierola, Mali (13.6583, -7.2155)			TB820A	Hymenoptera	Apocrita	Platygastroidea	Scelionidae	Scelioninae	Gryonini	*Gryon*		Elijah Talamas
Thierola, Mali (13.6	10-Aug-14	190	TB527A-HY-2	Hymenoptera	Apocrita	Platygastroidea	Scelionidae	Scelioninae		*Calliscelio*		Elijah Talamas
Thierola, Mali (13.6	24-Aug-14	190	TB563B-HY-3	Hymenoptera	Apocrita	Platygastroidea	Scelionidae	Scelioninae		*Dicroscelio*		Elijah Talamas
Thierola, Mali (13.6	24-Aug-14	190	TB563A-HY-2	Hymenoptera	Apocrita	Platygastroidea	Scelionidae	Scelioninae		*Fusicornia*	*eos*	Elijah Talamas
Thierola, Mali (13.6	24-Aug-14	190	TB563B-HY-3	Hymenoptera	Apocrita	Platygastroidea	Scelionidae	Teleasinae	Teleasini	*Trimorus*		Elijah Talamas
Markabougou, Mali	22-Aug-13	40	MB261A-HY-1	Hymenoptera	Apocrita	Vespoidea	Formicidae	Myrmicinae	Crematogastrini	*Crematogaster*		Brendon Boudinot
Suigima, Mali (14.1	2-Sep-14	190	SB419A-HY-1	Hymenoptera	Apocrita	Vespoidea	Formicidae	Myrmicinae	Crematogastrini	*Tetramorium*		Brendon Boudinot
Thierola, Mali (13.6	3-Aug-14		TB512A-HY-2	Hymenoptera	Apocrita	Vespoidea	Formicidae	Myrmicinae	Stenammini	*Messor*		Brendon Boudinot
Markabougou, Mali	14-Aug-13	160	MB242A-HY-3	Hymenoptera	Apocrita	Vespoidea	Formicidae	Ponerinae	Ponerini	*Anochetus*		Brendon Boudinot
Thierola, Mali (13.6	28-Oct-14	160	TB682A-HY-1	Hymenoptera	Apocrita	Vespoidea	Formicidae	Ponerinae	Ponerini	*Brachyponera*		Brendon Boudinot
Suigima, Mali (14.1	14-Aug-13	160	SB173A-HY-1	Hymenoptera	Apocrita	Vespoidea	Formicidae					
Thierola, Mali (13.6	5-Aug-13	40	TB372A-HY-2	Hymenoptera	Apocrita	Vespoidea	Pompilidae					
Thierola, Mali (13.6	3-Aug-14		TB512A-HY-2	Hymenoptera	Apocrita	Vespoidea	Rhopalosomatidae					Elijah Talamas
Thierola, Mali (13.6	2-Nov-15	120	TB1062A	Neuroptera	Hemerobiiformia	Chrysopoidea	Chrysopidae	Chrysopinae	Chrysopini	*Brinckochrysa*		Stephen J Brooks
Thierola, Mali (13.6	7-Jul-14	160	TB462B	Neuroptera	Hemerobiiformia	Chrysopoidea	Chrysopidae	Chrysopinae	Chrysopini	*Chrysoperla*	*congrua*	Stephen J Brooks
Suigima, Mali (14.1666, -7.2332)			SB113A	Neuroptera	Hemerobiiformia	Mantispoidea	Mantispidae					Laura Verú
Thierola, Mali (13.6	22-Jul-13	120	TB361A-OR-1	Orthoptera	Caelifera	Acridoidea	Acrididae	Gomphocerinae				Hojun Song
Thierola, Mali (13.6	22-Jul-13	120	TB361A-OR-1	Orthoptera	Caelifera	Acridoidea	Acrididae	Oedipodinae				Hojun Song
Thierola, Mali (13.6	5-Aug-13	40	TB372A-OR-2	Orthoptera	Caelifera	Acridoidea	Acrididae					Hojun Song
Thierola, Mali (13.6	23-Jul-13	120	TB364A-OR-1	Orthoptera	Caelifera	Pyrgomorphoidea	Pyrgomorphidae	Pyrgomorphinae	Atractomorphini	*Atractomorpha*		Ricardo Marino-Pe
Thierola, Mali (13.6	19-Jul-13	40	TB351A-OR-1	Orthoptera	Caelifera	Pyrgomorphoidea	Pyrgomorphidae	Pyrgomorphinae	Pyrgomorphini	*Pyrgomorpha*		Ricardo Marino-Pe
Thierola, Mali (13.6	13-Oct-15	190	TB1033A-OR-2	Orthoptera	Caelifera	Tetrigoidea	Tetrigidae	Tetriginae	Tetrigini			Hojun Song
Thierola, Mali (13.6	28-Oct-14	190	TB683A-OR-2	Orthoptera	Ensifera	Grylloidea	Gryllidae	Oecanthinae	Oecanthini	*Oecanthus*		Song Lab
Thierola, Mali (13.6	13-Oct-15	190	TB1033A-OR-1	Orthoptera	Ensifera	Tettigonioidea	Tettigoniidae	Conocephalinae				Derek A. Woller &
Thierola, Mali (13.6	12-Oct-14	120	TB633A-OR-1	Orthoptera	Ensifera	Tettigonioidea	Tettigoniidae	Phaneropterinae				Derek A. Woller &
Markabougou, Mali	7-Nov-14	160	MB493A-OR-1	Orthoptera	Ensifera	Tettigonioidea	Trigonidiidae	Trigonidiinae	Trigonidiini			Hojun Song
Thierola, Mali (13.6583, -7.2155)			TB349I	Neuroptera	Myrmeleontiformi	Myrmeleontoidea	Myrmeleontidae					Laura Verú
Thierola, Mali (13.6583, -7.2155)			TB502A	Thysanoptera								Laura Verú
Thierola, Mali (13.6583, -7.2155)							Culicidae					

Table S2. Taxonomical, ecological, and natural history of selected taxa.

Table S2: Identification, size, ecological services, and natural history of selected taxa

Taxon	Order	Family	Taxonomist	Body Length (	Mass (mg)	Ecological Service	Taxa affected	DietA:gen/spec	Habitat	HabitatLarvae	Aestivation	Generatime	Migration (genus level)
Dysdercus sp. Audinet-Serville	Hemiptera	Pyrrhocoridae	Thomas Henry	13.8	43.52	Agricultural Pest	Cotton and many other crops	seeds malvales generalist	Lush vegetation	Baobab/Cotton/malvales, even millet, sorghum			Windborne LDM in Wafrica: Ivory coast
Cysteochila endeca Drake	Hemiptera	Tingidae	Thomas Henry	3.23	0.57	Agricultural Pest	Pest of tamarind, rice	generalist					
Metacanthus nitidus Štusá	Hemiptera	Berytidae	Carsten Morkel	5.7	0.83		Feed on nettle: Fleuria aestuans may also be a predator of crop pests	Generalist	steppe and semi-desert				Suspected LDM
Nephotettix modulatus Melichar	Hemiptera	Cicadellidae	Charles Bartlett	4.56	2.98	Agricultural Pest	Voracious herbivore on crops and a vector of viral diseases	Generalist	Rice, millet, grasses	Rice, millet, grasses			
Anopheles coluzzii Coetzee & Wilkerson	Diptera	Culicidae	Adama Dao	1.2	1.5	Human Disease Vector	Primary vector of malaria and other human diseases	human specific	Standing water, peridomestic	aquatic	Yes: Dec-May	10-14 d	Windbone LDM in W Africa
Zolotarevskyella rhytidera (Chaudoir)	Coleoptera	Carabidae	Lourdes Chamorr	2.93	0.58	Predator of agricultural pests?	Predator agricultural pest species	Generalist					
Paederus sabeus Erichson	Coleoptera	Staphylinidae	Howard Frank	7.5	5.3	Agent of dermititis in people and a predator of agricultural pests	Medically important and predatory on crop pests	generalist predator	Wet habitats: marshes, edges of lakes, streams, rice fields	Wet habitats: marshes, edges of lakes, streams, rice fields			
Paederus fuscipes Curtis	Coleoptera	Staphylinidae	Howard Frank	6.3	3.32	Agent of dermititis in people and a predator of agricultural pests	Medically important and predatory on crop pests. Human infestations coincided with the local rice harvest	generalist predator	Wet habitats: marsh, edges of lakes, streams, rice fields	Wet habitats: marshes, edges of lakes, streams, rice fields		45 d @28C	
Chaetocnema coletta Bechyn	Coleoptera	Chrysomelidae	Maurizio Biondi	2.18	1.15	Agricultural Pest	Genus is a pest of corn, sweet potato, barley, wheat, sorghum, bean, alfalfa, rye, sugarbeet. Feeds voraciously on foliage; vector plant pathogen: RYMV	generalist?	Wet vegetation and montane grasslands, absent from desert areas	moist environments			
Berosus sp. Leach	Coleoptera	Hydrophilidae	Warren Steiner	3.05	1.7	Predators of mosquito larvae	Predator of mosquito larvae	Generalist	Aquatic: ponds, puddles, streams	Aquatic: ponds, puddles, streams	into soil enter domrancy		
Microchelonus sp. (Szépligeti)	Hymenoptera	Brachonidae	Elijah Talamas	3.16	1.02	Parasitoid of agricultural pests?	Unknown host, typically lepidopterans as Tortricidae, Gelechiidae and others	Specialist	Lepidopteran egg/caterpillar	host body			
Hypotrigona sp. Cockerell	Hymenoptera	Megachillidae	Corey Smith, John	3.19	2.74	Pollinator -high importance	Frequent crop visitors in E Africa, collect nectar and pollen from almost all crop plant species.	Generalist	well distributed in the tropics in various habitats. In East Africa: grasslands, natural forests, wetlands, farmlands,	Social bees nest in soils and wood. Uganda: commonly nest on walls of old buildings and in dry wood in forests. Sometimes in termite mounds.			
Hydrovatus sp. Motschulsky	Coleoptera	Dytiscidae	Saverio Rocchi	2.12	1.69	Predators of mosquito larvae	Predator of mosquito larvae	Predator	Aquatic: ponds, puddles, streams	Aquatic: ponds, puddles, streams			
